# Top 100 most-cited papers on diabetes mellitus in Dentistry: a bibliometric study

**DOI:** 10.1590/1807-3107bor-2024.vol38.0075

**Published:** 2024-08-05

**Authors:** Alexandre Henrique dos REIS-PRADO, Kiani dos Santos de PAULA, Gabriel Pereira NUNES, Lucas Guimarães ABREU, Luciano Tavares Angelo CINTRA, Isabella Faria da Cunha PEIXOTO, Francine BENETTI

**Affiliations:** (a) Universidade Federal de Minas Gerais – UFMG, School of Dentistry, Department of Restorative Dentistry, Belo Horizonte, MG, Brazil.; (b) Universidade Estadual Paulista – Unesp, School of Dentistry of Araçatuba Department of Restorative Dentistry, São Paulo, SP, Brazil.; (c) Universidade Federal de Minas Gerais – UFMG, School of Dentistry, Child’s and Adolescent’s Oral Health, Belo Horizonte, MG, Brazil.

**Keywords:** Bibliometrics, Databases, Bibliographic, Diabetes Mellitus, Hyperglycemia

## Abstract

This study assessed the features of the 100 most-cited papers on diabetes mellitus (DM) in dentistry using bibliometric measures. A search of the most cited papers on DM using journals included in the category “Dentistry, Oral Surgery and Medicine” in the Web of Science database up to January 2023 was performed. The complete bibliographic records of the selected papers were exported in plain text or Research Information Systems (RIS) file format. The following bibliometric indicators were collected: title, year, authors, number of citations, mean number of citations, institution, country, continent, study design, journal, impact factor, and keywords. Graphical bibliometric networks were created using the VOSviewer software. The number of citations for the 100 most-cited papers in DM research ranged from 111 to 566. Six papers each had more than 400 citations. Most were observational studies (n = 50) from the United States (USA) (n = 23) and were published in the *Journal of Periodontology* (30%; n=30). Robert Genco was the most cited author and contributed the most to the top 100 articles (3,653 citations; n = 13). The VOSviewer map of co-authorship showed the existence of clusters in research collaboration. The most prolific institutions were the Universities of Buffalo and Michigan (n = 6 each). “Diabetes mellitus” was the most frequent keyword, with 31 occurrences. In conclusion, the most cited studies that investigated the relationship between dentistry and DM were in periodontology. Observational studies, primarily from the USA, have been the most cited thus far.

## Introduction

Diabetes mellitus (DM) is a metabolic disease characterized by hyperglycemia, which affected 537 million individuals aged 20–79 years in 2021.^1,2^ DM results in elevated levels of inflammatory markers and increased susceptibility to infections,^3,4^ leading to progressive complications in various organs and tissues. This ultimately reduces quality of life and increases morbidity and mortality among patients.^5,6^

DM exhibits a spectrum of oral manifestations and/or complications, including a high prevalence and severity of dental caries and periapical bone resorption,^7,8^ impaired periodontal wound healing,^9,10^ and an elevated risk of developing oral potentially malignant disorders.^11^ A recent systematic review also showed that DM is associated with increased degeneration and mineralization within pulp tissue.^2^

Over the past 35 years, bibliometric analyses have measured the impact, trends, and development in various field of health research through quali-quantitative analyses of the number of citations of papers.^12^ Several bibliometric studies focusing on DM have been performed in the last three years, particularly within the medical sciences.^13-16^

The evaluation of oral alterations and correlation between different materials/therapies and systemic disorders, such as DM, has become an emerging field of interest in oral health research over time. However, to the best of our knowledge, no bibliometric analysis has been undertaken to comprehensively understand the hot topics, leading research centers, and potential future directions of DM research within dentistry.

In this context, a bibliometric analysis of the most-cited articles pertaining to DM in oral health research can help clinicians and researchers identify prominent authors, countries, journals, and institutions with high publishing activity, while also delineating research trends over time. Therefore, this study assessed the features of the 100 most-cited papers on DM in Dentistry through bibliometric measures, aiming to elucidate the impact and dissemination of different study designs on DM across the dental scientific community.

## Methods

### Information sources and search strategy

In January 2023, a bibliometric analysis focused on the 100 most cited papers on DM research in Dentistry was conducted in the Web of Science “All Databases” (WoS-AD) and “Core Collection” (WoS-CC) in the category of ‘Dentistry, Oral Surgery, and Medicine’. The following search terms were used: (“diabetes mellitus” OR diabetes OR diabetic OR diabetics OR “diabetic mellitus” OR “diabetics mellitus” OR “type 2 diabetes mellitus” OR “type 1 diabetes mellitus” OR “insulin resistance” OR “insulin sensitivity” OR” high glucose” OR hyperglycemic OR hyperglycaemia OR hyperglycemia OR “glycated hemoglobin” OR “diabetes complications”).

### Study selection and data collection

Three researchers (A.H.R.P., G.P.N. and K.S.P.) independently conducted the study selection based on eligibility criteria. These authors reviewed the title and abstracts of the identified articles, and if necessary, conducted full-text reading. The resulting list was arranged in descending order of WoS citations. The three researchers also undertook data extraction. During screening of the articles identified, restrictions on language or year of publication were not imposed in any way. Any disagreements on study selection and data extraction were resolved through discussion and consensus. Papers whose main focus was not related to DM or those that did not present at least a dedicated topic were excluded. Likewise, letters to the editor and reports on meeting abstracts were also excluded. In cases where more than one paper had the same number of citations, the more recently published paper received a higher rank. The assessment concluded upon finding the 100^th^ most-cited paper. Complete bibliographic records of the selected papers were exported in plain text or Research Information Systems (RIS) file format from WoS and imported into the VOSviewer software (version 1.6.7; Leiden University Center for Science and Technology Studies, Leiden, Netherlands) for statistical computation and graphics.

The following information was extracted from each paper: title of the article; year of publication; first and others authors; number of citations; the citation mean per year (ratio of the numbers of citations and the period since the year of publication until December 2022); research center or institution/university; country and continent based on corresponding author’s affiliation; study design; journal and Journal Citation Reports Impact Factor (JCR^®^ IF 2021) in the WoS subject category “Dentistry, Oral Surgery & Medicine” for the year in which the papers had been published; and finally, the keywords. Study designs were classified as follows: literature review, laboratorial studies *(in vitro, in vivo, in situ, ex vivo*), observational study, randomized controlled trial (RCT), non-randomized clinical study, and systematic review with or without meta-analysis.

### Data analysis and visualization

The VOSviewer software was used to generate bibliometric networks. Within these maps, authors’ names were pooled into the VOSviewer as a unit of analysis and were linked to each other based on the number of co-authored papers. A collaboration network was constructed for co-authors who had contributed to three or more articles.^17^ In the networks, clusters consisted of groups of nodes that were closely related, with each cluster assigned a specific color. The node size indicated the total number of articles published by each co-author. Larger circles indicated more relevant terms, and strongly related terms were positioned closer to each other. Furthermore, lines between terms indicated existing relationships, with thicker lines representing stronger connections between two items.^18^

## Results

The screening process identified 12,436 papers from WoS-AD classified under the “Dentistry, Oral Surgery and Medicine” category. Following the ranking of this list in descending order of citation count, 132 papers were excluded due to their lack of focus on the evaluated field. The top 100 most-cited papers, along with their respective citation counts, are presented in Table 1.

**Table 1:** The top 100 most-cited papers on diabetes mellitus published in Dentistry.

Rank	Title	Year of publication	First author	Other authors	Web of Science - All databases	Web of Science - Core Collection	Institution	Country	Continent	Study design	Journal (JCR® IF 2021)	Keywords
1	Diabetes mellitus and periodontal diseases	2006	Mealey BL	Oates TW	566 (35.37)	532 (33.25)	University of Texas	USA	America	Literature review	Journal of Periodontology (4.494)	Diabetes mellitus; inflammation; insulin resistance; obesity; periodontal diseases
2	Diabetes and periodontal diseases: consensus report of the Joint EFP/AAP on Periodontitis and Systemic Diseases	2013	Chapple ILC	Genco R	470 (52.22)	448 (49.77)	University of Birmingham	England	Europe	Observational study	Journal of Periodontology (4.494)	Association; complications; diabetes mellitus; gestational diabetes; HbA1C; incident; intervention; mechanisms; periodontal disease; periodontitis; type 2 diabetes
3	The role of inflammatory cytokines in diabetes and its complications	2008	King GL	n.a.	468 (33.42)	427 (30.5)	Harvard University	USA	America	Literature review	Journal of Periodontology (4.494)	Complications; diabetes; hyperglycemia; inflammation; insulin resistance; periodontal disease
4	Evidence for cigarette-smoking as a major risk factor for periodontitis	1993	Haber J	Wattles J, Crowley M, Mandell R, Joshipera K, Kent RL	443 (15.27)	423 (14.58)	Tufts University	USA	America	Observational study	Journal of Periodontology (4.494)	Tobacco adverse effects; diabetes-mellitus; risk factors
5	A proposed model linking inflammation to obesity, diabetes, and periodontal infections	2005	Genco RJ	Grossi SG, Ho A, Nishimura F, Murayama Y	439 (25.82)	427 (25.11)	SUNY Buffalo State University	USA	America	Observational study	Journal of Periodontology (4.494)	Diabetes; insulin resistance; obesity; periodontal disease; soluble tumor necrosis factor receptor; TNF-alpha
6	Periodontal disease in non-insulin dependent diabetes mellitus	1991	Emrich LJ	Shlossman M, Genco RJ	423 (13.64)	405 (13.06)	SUNY Buffalo State University	USA	America	Observational study	Journal of Periodontology (4.494)	Indians; north american; diabetes; mellitus; oral health index;periodontal diseases epidemiology; periodontal diseases etiology
7	Severe periodontitis and risk for poor glycemic control in patients with non-insulin-dependent diabetes mellitus	1996	Taylor GW	Burt BA, Becker MP, Genco RJ, Shlossman M, Knowler WC, Pettit DJ	377 (14.5)	360 (13.84)	University of Michigan	USA	America	Observational study	Journal of Periodontology (4.494)	Periodontitis complications; diabetes mellitus, non-insulin dependent; hyperglycemia; hypoglycemia; longitudinal studies; epidemiology; models statistical
8	Minocycline reduces gingival collagenolytic activity during diabetes - preliminary-observations and a proposed new mechanism of action	1983	Golub LM	Lee HM, Lehrer G, Nemiroff A, Mcnamara TF, Kaplan R, Ramamurthy NS	370 (9.48)	357 (9.15)	Stony Brook University	USA	America	In vivo	Journal of Periodontal Research (3.946)	n.a.
9	Periodontal disease: associations with diabetes, glycemic control and complications	2008	Taylor GW	Borgnakke WS	339 (24.21)	309 (22.07)	University of Michigan	USA	America	Literature review	Oral Diseases (4.068)	Periodontal disease; diabetes; epidemiology; periodontal treatment
10	Treatment of periodontal disease in diabetics reduces glycated hemoglobin	1997	Grossi SG	Skrepcinksi FB, DeCaro T, Robertson DC, Ho AW, Dunford RG, Genco RJ	333 (13.32)	315 (12.6)	SUNY Buffalo State University	USA	America	Randomized controlled trial	Journal of Periodontology (4.494)	Diabetes mellitus, non-insulin dependent, epidemiology; periodontal diseases, therapy; doxycycline, therapeutic use; chlorhexidine, therapeutic use; povidoneiodine; therapeutic use; Porphyromonas gingivalis
11	Glycemic control of type 2 diabetes and severe periodontal disease in the US adult population	2002	Tsai C	Hayes C, Taylor GW	326 (16.3)	305 (15.3)	University of Michigan	USA	America	Observational study	Community Dentistry and Oral Epidemiology (2.489)	Adults; diabetes mellitus; epidemiology; glycosylated hemoglobin; logistic models; periodontal diseases; type 2 diabetes mellitus; United States
12	Effect of periodontal disease on diabetes: systematic review of epidemiologic observational evidence	2013	Borgnakke WS	Ylostalo PV, Taylor GW, Genco RJ	324 (36)	314 (34.88)	University of Michigan	USA	America	Systematic review	Journal of Periodontal Research (3.946)	Diabetes complications; diabetes mellitus; gestational diabetes; epidemiology; haemoglobin a; glycosylated; humans; periodontal diseases; review; systematic
13	Global burden of oral diseases: emerging concepts, management and interplay with systemic health	2016	Jin LJ	Lamster IB, Greenspan JS, Pitts NB, Scully C	289 (48.1)	278 (46.33)	University of Hong Kong	Hong Kong	Asia	Literature review	Oral Diseases (4.068)	Disease burden; oral diseases; non-communicable diseases; common risk factors approach; caries; periodontal disease; edentulism; oral cancer; oral mucosal diseases; cleft lip; palate
14	Prevalence of periodontal disease in insulin-dependent diabetes mellitus (juvenile diabetes)	1982	Canciola LJ	Park BH, Bruck E, Mosovich, L, Genco, RJ	285 (7.12)	278 (6.95)	SUNY Buffalo State University	USA	America	Observational study	Journal of the American Dental Association (3.454)	n.a.
15	Diabetes mellitus and periodontal diseases	2007	Mealey BL	Ocampo GL	269 (17.93)	250 (16.66)	n.i.	n.i.	n.i.	Literature review	Periodontology 2000 (12.239)	n.a.
16	The effect of improved periodontal health on metabolic control in type 2 diabetes mellitus	2005	Kiran M	Arpak N, Unsal E, Erdogan MF	246 (14.47)	223 (13.11)	Ankara University	Turkey	Asia	Observational study	Journal of Clinical Periodontology (7.478)	Metabolic control; periodontal therapy; type 2 diabetes mellitus
17	Scientific evidence on the links between periodontal diseases and diabetes: Consensus report and guidelines of the joint workshop on periodontal diseases and diabetes by the International Diabetes Federation and the European Federation of Periodontology	2018	Sanz M	Sanz M, Ceriello A, Buysschaert M, Chapple I, Demmer RT, Graziani F, Herrera D, Jepsen S, Lione L, Madianos P, Mathur M, Montanya E, Shapira, Tonetti M, Vegh D	243 (60.75)	240 (60)	Complutense University of Madrid	Spain	Europe	Literature review	Journal of Clinical Periodontology (7.478)	Association; chronic kidney disease; complications; diabetes mellitus; gestational diabetes; HbA1c; incident; intervention; mechanisms; mortality; nephropathy; periodontal disease; periodontitis; retinopathy; type 1 diabetes; type 2 diabetes
18	The relationship between oral health and diabetes mellitus	2008	Lamster, IB	Lalla E, Borgnakke WS, Taylor GW	242 (17.28)	236 (16.85)	Columbia University	USA	America	Literature review	Journal of the American Dental Association (3.454)	Diabetes mellitus; oral health; oral candidiasis; periodontitis
19	The relationship between diabetes mellitus and destructive periodontal disease: A meta-Analysis	2009	Chavarry NGM	Vettore MV, Sansone C, Sheiham A	235 (18.07)	226 (17.38)	Federal University of Rio de Janeiro	Brazil	America	Systematic review/meta-analysis	Oral Health and Preventive Dentistry (1.595)	Diabetes mellitus; meta-analysis; periodontal disease; periodontitis; systematic review
20	Type 2 diabetes mellitus and periodontal disease	1990	Shlossman M	Knowler WC, Pettitt DJ, Genco RJ	223 (6.96)	210 (6.56)	SUNY Buffalo State University	USA	America	Observational study	Journal of the American Dental Association (3.454)	n.a.
21	The RANKL-OPG system in clinical periodontology	2012	Belibasakis GN	Bostanci N	220 (22)	206 (20.6)	University of Zürich	Switzerland	Europe	Literature review	Journal of Clinical Periodontology (7.478)	Diagnostics; gingival crevicular fluid; osteoprotegerin; periodontal disease; receptor activator of NF-?B ligand; receptor activator of NF-?B ligand; osteoprotegerin ratio
22	Comparison of periodontal and socioeconomic status between subjects with type 2 diabetes Mellitus and non-diabetic controls	2007	Javed F	Nasstrom K, Benchimol D, Altamash M, Klinge B, Engstrom PE	212 (14.13)	207 (13.8)	Karolinska Instute	USA	America	Observational study	Journal of Periodontology (4.494)	Bleeding; bone loss; smoking; socioeconomic status; type 2 diabetes
23	Impact of diabetes mellitus and glycemic control on the osseointegration of dental implants: A systematic literature review	2009	Javed F	Romanos GE	210 (16.15)	205 (15.76)	Karolinska Institute	Sweden	Europe	Systematic review	Journal of Periodontology (4.494)	Dental implants; diabetes mellitus; hyperglycemia; osseointegration; periodontal bone loss
24	Diabetes enhances periodontal bone loss through enhanced resorption and diminished bone formation	2006	Liu R	Bal HS Desta T, Krothapalli N, Alyassi M, Luan Q, Graves DT	207 (12.93)	184 (11.5)	Boston University	USA	America	In vivo	Journal of Dental Research (8.924)	Bone coupling; cell death; bacteria; gingiva; hyperglycemia; inflammation; infection; in vivo; periodontitis
25	Does periodontal treatment improve glycemic control in diabetic patients? A meta-analysis of intervention studies	2005	Janket SJ	Wightman A, Baird AE, Van Dyke TE, Jones JA	202 (11.88)	194 (11.41)	Boston University	USA	America	Systematic review/meta-analysis	Journal of Dental Research (8.924)	Meta-analysis; inflammatory mediators; hemoglobin A1c; non-surgical periodontal treatment; antibiotics treatment
26	Response to periodontal therapy in diabetics and smokers	1996	Grossi SG	Skrepcinski FB, DeCaro T, Zambon JJ, Cummins D, Genco RJ,	202 (7.76)	189 (7.26)	State University of New York	USA	America	Observational study	Journal of Periodontology (4.494)	Host response; periodontal diseases therapy; risk factors; smoking adverse effects; wound healing; diabetes mellitus complications
27	Advanced glycation endproducts (AGEs) induce oxidant stress in the gengiva: A potential mechanism underlying accelerated periodontal disease associated with diabetes	1996	Schmidt AM	Weidman, E, Lalla E, Yan SD, Hori O, Cao R, Brett JG, Lamster IB	198 (7.61)	189 (7.26)	Columbia University	USA	America	In vivo	Journal of Periodontal Research (3.946)	Glycation; oxidation; diabetes; periodontum
28	Inflammatory mediator response as a potential risk marker for periodontal diseases in insulin-dependent diabetes mellitus patients	1997	Salvi GE	Yalda B, Collins JG, Jones BH, Smith FW Arnold RR, Offenbacher S	194 (7.76)	184 (7.36)	University of North Carolina	USA	America	Observational study	Journal of Periodontology (4.494)	Gingival crevicular fluid analysis; diabetes mellitus; prostaglandin E(2); interleukin 1 beta
29	Non-insulin dependent diabetes mellitus and alveolar bone loss progression over 2 years	1998	Taylor GW	Burt BA, Becker MP, Genco RJ, Shlossman M, Knowler WC, Pettit DJ	191 (7.95)	178 (7.41)	University of Michigan	USA	America	Observational study	Journal of Periodontology (4.494)	Periodontitis; diabetes mellitus; diabetes mellitus, non-insulin dependent; longitudinal studies; epidemiology; alveolar bone loss
30	The effect of antimicrobial periodontal treatment on circulating tumor necrosis factor-alpha and glycated hemoglobin level in patients with type 2 diabetes	2001	Iwamoto Y	Nishimura F, Nakagawa M, Sugimoto H, Shikata K, Makino, H, Fukuda, T, Tsuji T, Iwamoto M, Murayama Y	189 (9)	176 (8.3)	Okayama University	Japan	Asia	Non-randomized clinical study	Journal of Periodontology (4.494)	Diabetes mellitus; non-insulin dependent/prevention and control; insulin resistance; obesity in diabetes; periodontal diseases/therapy; tumor necrosis factor; minocycline/therapeutic use
31	Periodontal disease and diabetes - A two-way street	2006	Mealey, BL	n.a.	185 (11.56)	178 (11.12)	University of Texas	USA	America	Literature review	Journal of the American Dental Association (3.454)	Diabetes mellitus; periodontal diseases; periodontal therapy; inflammation
32	The effect of periodontal treatment on glycemic control in patients with type 2 diabetes mellitus	2001	Stewart JE	Wager KA, Friedlander AH, Zadeh HH	184 (8.76)	170 (8.1)	Greater Los Angeles Health Care System	USA	America	Randomized controlled trial	Journal of Clinical Periodontology (7478)	Adult periodontitis; root planing; subgingival curettage; type 2 diabetes mellitus; glycemic control; hemoglobin A(1C)
33	Severe periodontitis is associated with systemic inflammation and a dysmetabolic status: a case-control study	2007	Nibali L	D’Aiuto F, Griffiths G, Patel K, Suvan J, Tonetti MS	183 (12.2)	172 (11.46)	University College London	England	Europe	Observational study	Journal of Clinical Periodontology (7.478)	Cardiovascular disease; dyslipidemia; insulin resistance; metabolic syndrome; periodontitis; systemic inflammation
34	Plasma lipid and blood glucose levels in patients with destructive periodontal disease	2000	Lösche W	Karapetow F, Pohl A, Pohl C, Kocher T	178 (8.1)	171 (7.8)	University of Jena	Germany	Europe	Observational study	Journal of Clinical Periodontology (7.478)	Periodontitis; cardiovascular disease; plasma lipids; blood glucose; risk factors
35	Prevalence and predictive factors for peri-implant disease and implant failure: A cross-sectional analysis	2015	Daubert DM	Weinstein BF, Bordin S, Leroux BG, Flemmig TF	174 (24.85)	171 (24.42)	University of Washington	USA	America	Observational study	Journal of Periodontology (4.494)	Dental implants; diabetes mellitus; follow-up studies; peri-implantitis; periodontitis; risk factors
36	Type 1 diabetes mellitus, xerostomia, and salivary flow rates	2001	Moore PA	Guggenheimer J, Etzel KR, Weyant RJ, Orchard T	174 (8.3)	164 (7.81)	University of Pittsburgh	USA	America	Observational study	Oral Surgery, Oral Medicine, Oral Pathology, Oral Radiology, and Endodontology (2.538)	n.a.
37	Obesity, diabetes mellitus, atherosclerosis and chronic periodontitis: a shared pathology via oxidative stress and mitochondrial dysfunction?	2014	Bullon P	Newman HN, Battino M	167 (20.9)	158 (19.8)	n.i.	n.i.	n.i.	Literature review	Periodontology 2000 (12.239)	n.a.
38	Periodontal disease and diabetes mellitus: The role of tumor necrosis factor-alpha in a 2-way relationship	2003	Nishimura F	Iwamoto Y, Mineshiba J, Shimizu A, Soga Y, Murayama Y	167 (8.8)	157 (8.26)	Okayama University	Japan	Asia	Case report	Journal of Periodontology (4.494)	Diabetes mellitus; type 2; insulin resistance; obesity; periodontal diseases/etiology; risk factors; tumor necrosis factor
39	Effect of non-surgical periodontal therapy on glycemic control in patients with type 2 diabetes mellitus	2003	Rodrigues DC	Taba M, Novaes AB, Souza SLS, Grisi MFM	165 (8.68)	152 (8)	University of São Paulo	Brazil	America	Randomized controlled trial	Journal of Periodontology (4.494)	Comparison studies; diabetes mellitus; non-insulin dependent; hemoglobin; glycated; periodontal diseases/drug therapy; periodontal diseases/therapy
40	Healing response to non-surgical periodontal therapy in patients with diabetes mellitus: clinical, microbiological, and immunologic results	1998	Christgau M	Palitzsch KD, Schmalz G, Kreiner U, Frenzel S	164 (6.83)	158 (6.58)	University of Texas	USA	America	Observational study	Journal of Clinical Periodontology (7.478)	Diabetes mellitus; diabetes complications; periodontal disease/therapy; periodontal disease/microbiology; risk factors; neutrophils
41	Evidence that periodontal treatment improves diabetes outcomes: a systematic review and meta-analysis	2013	Engebretson S	Kocher T	163 (18.1)	158 (17.55)	New York University	USA	America	Systematic review/meta-analysis	Journal of Clinical Periodontology (7.478)	Diabetes; diabetes mellitus; glycosylated haemoglobin; HbA1c; periodontal disease; periodontitis; type 2
42	How do smoking, diabetes, and periodontitis affect outcomes of implant treatment?	2007	Klokkevold PR	Han TJ	163 (10.9)	161 (10.73)	University of California	USA	America	Systematic review	The International Journal of Oral & Maxillofacial Implants (2.912)	Dental implants; dental implant survival; diabetes; periodontitis; smoking; tobacco
43	Relationship between obesity, glucose tolerance, and periodontal disease in Japanese women: the Hisayama study	2005	Saito T	Shimazaki Y, Kiyohara Y, Kato I, Kubo M, Iida M	163 (9.6)	158 (9.3)	Kyushu University	Japan	Asia	Observational study	Journal of Periodontal Research (3.946)	Diabetes; epidemiology; glucose tolerance; obesity; periodontal disease; risk factor
44	Diabetes and periodontal disease: A case-control study	2005	Campus G	Salem A, Uzzau S, Baldoni E, Tonolo G	163 (9.58)	148 (8.7)	University of Sassari	Italy	Europe	Observational study	Journal of Periodontology (4.494)	Diabetes; non-insulin dependent; periodontal diseases; risk factors; Sardinia
45	Metabolic syndrome and periodontitis: Is oxidative stress a common link?	2009	Bullon P	Morillo JM, Ramirez-Tortosa MC, Quiles JL, Newman HN, Battino M	162 (12.46)	153 (11.76)	Marche Polytechnic University	Italy	Europe	Literature review	Journal of Dental Research (8.924)	Metabolic syndrome; oxidative stress; periodontitis; hypertension; dyslipidemia; insulin resistance
46	Effects of diabetes mellitus on periodontal and peri-implant conditions: update on associations and risks	2008	Salvi GE	Carollo-Bittel B, Lang NP	158 (11.28)	150 (10.71)	University of Bern	Switzerland	Europe	Literature review	Journal of Clinical Periodontology (7.478)	Diabetes; glycaemic control; host response; inflammation; oral implants; periodontal disease; periodontal therapy; periodontitis
47	A systematic review and meta-analysis of epidemiologic observational evidence on the effect of periodontitis on diabetes An update of the EFP-AAP review	2018	Graziani F	Gennai S, Solini A, Petrini M	156 (39)	147 (36.75)	University of Pisa	Italy	Europe	Systematic review/meta-analysis	Journal of Clinical Periodontology (7.478)	Diabetes; epidemiology; periodontitis
48	Diabetes and periodontal disease: a two-way relationship	2014	Casanova L	Hughes FJ, Preshaw PM	155 (19.37)	143 (18)	Newcastle University	England	Europe	Literature review	British Dental Journal (2.727)	n.a.
49	The relationship between reduction in periodontal inflammation and diabetes control: a report of 9 cases	1992	Miller LS	Manwell MA, Newbold D, Reding ME, Rasheed A, Blodgett J, Kornman KS	155 (5.16)	140 (4.7)	University of Texas Health Science Center	USA	America	Observational study	Journal of Periodontology (4.494)	Diabetes-mellitus epidemiology; diabetes-mellitus etiology; blood glucose; periodontitis epidemiology; periodontitis therapy; risk factors
50	Patients with burning mouths. A clinical investigation of causative factors, including the climacteric and diabetes	1978	Basker RM	Sturdee DW, Davenport JC	154 (3.5)	149 (3.38)	University of Birmingham	England	Europe	Observational study	British Dental Journal (2.727)	n.a.
51	Metabolic disorders related to obesity and periodontal disease	2007	Saito T	Shimazaki Y	153 (10.2)	149 (9.93)	n.i.	n.i.	n.i.	Literature review	Periodontology 2000 (12.239)	n.a.
52	Long-term control of diabetes mellitus and periodontitis	1993	Tervonen T	Oliver RC	151 (5.20)	133 (4.58)	University of Oulu	Finland	Europe	Observational study	Journal of Clinical Periodontology (7.478)	
53	An update on the evidence for pathogenic mechanisms that may link periodontitis and diabetes	2018	Polak D	Shapira L	148 (37)	136 (34)	The Hebrew University of Jerusalem	Israel	Asia	Literature review	Journal of Clinical Periodontology (7.478)	Cytokines; diabetes; hyperglycemia; inflammation; periodontitis
54	Medical status and complications in relation to periodontal disease experience in insulin-dependent diabetics	1996	Thorstensson H	Kuylenstierna J, Hugoson A	146 (5.6)	141 (5.4)	Institute for Postgraduate Dental Education	Sweden	Europe	Observational study	Journal of Clinical Periodontology (7.478)	Diabetes mellitus; insulin-dependent diabetes; long-duration diabetes; medical complications; periodontal disease
55	The effect of diabetes mellitus on osseous healing	2010	Retzepi M	Donos N	145 (12.08)	140 (11.7)	University College London	England	Europe	Literature review	Clinical Oral Implants Research (5.021)	Bone healing; diabetes mellitus; hyperglycaemia; insulin; ossification
56	Monocytic TNF alpha secretion patterns in IDDM patients with periodontal diseases	1997	Salvi GE	Collins JG, Yalda B, Arnold RR, Lang NP, Offenbacher S	145 (6.59)	140 (6.36)	University of North Carolina	USA	America	Observational study	Journal of Clinical Periodontology (7.478)	Gram-negative bacterial infections; TNF alpha; monocytes; insulin-dependent diabetes mellitus (IDDM)
57	Expression of the receptor of advanced glycation end products in gingival tissues of type 2 diabetes patients with chronic periodontal disease: a study utilizing immunohistochemistry and RT-PCR	2005	Katz J	Bhattacharyya I, Farkhondeh-Kish F, Perez FM, Caudle RM, Heft MW	144 (8.47)	139 (8.17)	University of Florida	USA	America	In vivo	Journal of Clinical Periodontology (7.478)	Gingiva; receptor of advanced glycation end products; type 2 diabetes
58	The severity of periodontal disease is associated with the development of glucose intolerance in non-diabetics: The Hisayama study	2004	Saito T	Shimazaki Y, Kiyohara Y, Kato I, Kubo M, Iida M, Koga T	144 (8)	137 (7.61)	Kyushu University	Japan	Asia	Observational study	Journal of Dental Research (8.924)	Periodontal disease; diabetes; glucose tolerance; risk factor; epidemiology
59	Periodontal conditions in insulin-dependent diabetics	1989	Hugoson A	Thorslensson H, Falk H, Kuylenslierna J	143 (4.33)	140 (4.24)	Institute for Postgraduate Dental Education	Sweden	Europe	Observational study	Journal of Clinical Periodontology (7.478)	n.a.
60	Expression of matrix metalloproteinases (MMP-8 and -9) in chronic periodontitis patients with and without diabetes mellitus	2006	Kumar MS	Vamsi G, Sripriya R, Sehgal PK	142 (8.9)	138 (8.62)	Central Leather Research Institute	India	Asia	Non-randomized clinical study and in vivo	Journal of Periodontology (4.494)	Chronic periodontitis; collagen; diabetes mellitus; matrix metalloproteinases
61	Diabetes-A risk factor for periodontitis in adults?	1994	Oliver RC	Tervonen T	141 (5.03)	129 (4.6)	University of Minnesota	USA	America	Literature review	Journal of Periodontology (4.494)	Periodontitis etiology; diabetes-mellitus prevention and control; risk factors
62	Microbiological and immunological studies of adult periodontitis in patients with noninsulin-dependent diabetes mellitus	1988	Zambon JJ	Reynolds H, Fisher JG, Shlossman M, Dunford R, Genco RJ	141 (4.14)	138 (4.05)	SUNY Buffalo State University	USA	America	In vitro	Journal of Periodontology (4.494)	n.a.
63	Periodontal diseases and health: Consensus Report of the Sixth European Workshop on Periodontology	2008	Kinane D	Bouchard P	140 (10)	130 (9.28)	University of Louisville	USA	America	Literature review	Journal of Clinical Periodontology (7.478)	Cardiovascular; diabetes mellitus; periodontitis; preterm birth; prevalence
64	Relationship of metabolic syndrome to periodontal disease in Japanese women: The Hisayama study	2007	Shimazaki Y	Saito T, Yonemoto K, Kiyohara Y, Iida M, Yamashita Y	139 (9.26)	133 (8.9)	Kyushu University	Japan	Asia	Observational study	Journal of Dental Research (8.924)	Metabolic syndrome; periodontal disease; risk factor; epidemiology; Japanese women
65	Commonality in chronic inflammatory diseases: periodontitis, diabetes, and coronary artery disease	2006	Southerland JH	Taylor GW, Moss K, Beck JD, Offenbacher S	137 (8.56)	125 (7.81)	University of North Carolina	USA	America	Literature review	Periodontology 2000 (7.589)	n.a.
66	Systemic diseases affecting osseointegration therapy	2006	Mombelli A	Cionca N	136 (8.5)	134 (8.37)	University of Geneva	Switzerland	Europe	Literature review	Clinical Oral Implants Research (5.021)	Diabetes; implant failure; osteoporosis; systemic disease; osseointegration
67	Wound healing around endosseous implants in experimental diabetes	1998	Nevins ML	Karimbux NY, Weber HP, Giannobile WV, Fiorellini JP	134 (9.57)	126 (9)	Harvard University	USA	America	In vivo	The International Journal of Oral & Maxillofacial Implants (2.804)	Dental implants; diabetes; osseointegration; wound healing
68	Impact of local and systemic factors on the incidence of failures up to abutment connection with modified surface oral implants	2008	Alsaadi G	Quirynen M, Michiles K, Teughels W, Komárek A, van Steenberghe D	133 (9.5)	128 (9.14)	Catholic University of Leuven	Belgium	Europe	Observational study	Journal of Clinical Periodontology (7.478)	Dental implants; oral implants; osseointegration; systemic disease; TiUnite
69	Effect of non-surgical periodontal therapy on clinical and immunological response and glycaemic control in type 2 diabetic patients with moderate periodontitis	2007	Navarro-Sanchez AB	Faria-Almeida R, Bascones-Martinez A	133 (8.87)	124 (8.27)	Complutense University of Madrid	Spain	Europe	Observational study	Journal of Clinical Periodontology (7.478)	Cytokines; diabetes mellitus; metabolic control; periodontitis
70	Subgingival biodiversity in subjects with uncontrolled type-2 diabetes and chronic periodontitis	2013	Casarin RC	Barbagallo A, Meulman T, Santos VR, Sallum EA, Nociti FH, Duarte PM, Casati MZ, Gonçalves RB	132 (14.67)	117 (13)	Universidade Paulista	Brazil	America	Observational study	Journal of Periodontal Research (3.946)	Diabetes; periodontal microbiota; subgingival plaque
71	Diabetes mellitus related bone metabolism and periodontal disease	2015	Wu YY	Xiao E, Graves DT	129 (18.43)	119 (17)	University of Pennsylvania	USA	America	Literature review	International Journal of Oral Science (6.344)	Bone loss; diabetes mellitus; hyperglycemia; inflammation; osseous; osteoblast; osteoclast; periodontitis
72	A cohort study on the association between periodontal disease and the development of metabolic syndrome	2010	Morita T	Yamazaki Y, Mita A, Takada K, Seto M, Nishinoue N, Sasaki Y, Motohashi M, Maeno M.	129 (10.75)	126 (10.5)	Nihon University School of Dentistry	Japan	Asia	Observational study	Journal of Periodontology (4.494)	Cohort study; hyperglycemia; hypertension; lipid metabolism; obesity; periodontal disease
73	Diabetes and oral implant failure: a systematic review	2014	Chrcanovic BR	Albrektsson T	128 (16)	117 (14.63)	Malmö University	Sweden	Europe	Systematic review/meta-analysis	Journal of Dental Research (8.924)	Diabetes mellitus; blood glucose; dental implants; infection; periodontal bone loss; meta-analysis
74	A longitudinal study on insulin-dependent diabetes mellitus and periodontal disease	1993	Seppälä B	Seppälä M, Ainamo J	128 (4.41)	124 (4.28)	University of Helsinki	Finland	Europe	Observational study	Journal of Clinical Periodontology (7.478)	Periodontal diseases; longitudinal studies; insulin-dependent diabetes-mellitus
75	The Oral Microbiota Is Modified by Systemic Diseases	2019	Graves DT	Corrêa JD, Silva TA	127 (42.33)	122 (40.67)	University of Pennsylvania	USA	America	Literature review	Journal of Dental Research (8.924)	Bacteria; biofilm; dysbiosis; periodontitis; periodontium; inflammation
76	Diabetes-enhanced inflammation and apoptosis--impact on periodontal pathology	2006	Graves DT	Liu R, Alikhani M, Al-Mashat H, Trackman PC	126 (7.88)	117 (7.31)	Boston University	USA	America	Literature review	Journal of Dental Research (8.924)	Bacteria; bone; connective tissue; cytokine; cell death; diabetes; gingiva; hyperglycemia; infection; inflammatory; oral; periodontitis
77	Gingival crevicular fluid levels of interleukin-1beta and glycemic control in patients with chronic periodontitis and type 2 diabetes3	2004	Engebretson SP	Hey-Hadavi J, Ehrhardt FJ, Hsu D, Celenti SR, Grbic JT, Lamster IB	126 (7)	120 (6.67)	Columbia University	USA	America	Observational study	Journal of Periodontology (4.494)	Diabetes mellitus; non-insulin dependent; gingival crevicular fluid/chemistry; hemoglobin A, glycosylated; interleukin-1; periodontitis/etiology
78	Insulin-dependent diabetes mellitus and oral soft tissue pathologies: II. Prevalence and characteristics of Candida and Candidal lesions	2000	Guggenheimer J	Moore PA, Rossie K, Myers D, Mongelluzzo MB, Bloco HM, Weyant R, pomar T	126 (5.73)	112 (5.09)	University of Pittsburgh	USA	America	Observational study	Oral Surgery, Oral Medicine, Oral Pathology, and Oral Radiology (2.538)	n.a.
79	SARS-CoV-2, uncontrolled diabetes and corticosteroids-An unholy trinity in invasive fungal infections of the maxillofacial region? A retrospective, multi-centric analysis	2021	Moorthy A	Gaikwad R, Krishna S, Hegde R, Tripathi KK, Kale PG, Rao PS, Haldipur D, Bonanthaya K	124 (124)	124 (124)	Rangadore Memorial Hospital	India	Asia	Observational study	Journal of Maxillofacial and Oral Surgery (1.08)	Covid-19; Mucormycosis; Rhino-cerebro-orbital; Diabetes; Aspergillosis
80	The periodontal microflora of juvenile diabetics. Culture, immunofluorescence, and serum antibody studies	1983	Mashimo PA	Yamamoto Y, Slots J, Park BH, Genco RJ	124 (3.18)	123 (3.15)	New York University	USA	America	Literature review	Journal of Periodontology (4.494)	n.a.
81	Identification of unrecognized diabetes and pre-diabetes in a dental setting	2011	Lalla E	Kunzel C, Burkett S, Cheng B, Lamster IB	123 (11.18)	119 (10.82)	Columbia University	USA	America	Observational study	Journal of Dental Research (8.924)	Diabetes; periodontal disease(s)/periodontitis; periodontal medicine; hyperglycemia; undiagnosed; screening
82	The effect of diabetes mellitus on endodontic treatment outcome: data from an electronic patient record	2003	Fouad AF	Burleson J	123 (6.47)	118 (6.21)	University of Connecticut Health Center	USA	America	Observational study	Journal of the American Dental Association (3.634)	n.a.
83	Periodontal disease as a potential risk factor for systemic diseases	1998	Scannapieco FA	n.a.	122 (5.08)	112 (4.67)	State University of New York	USA	America	Literature review	Journal of Periodontology (4.494)	n.a.
84	Clinical and public health implications of periodontal and systemic diseases: An overview	2020	Genco RJ	Sanz M	121 (60.5)	115 (57.5)	Complutense University of Madrid	Spain	Europe	Systematic review	Periodontology 2000 (7.589)	Cardiovascular diseases; chronic non-communicable diseases; diabetes; intervention studies; periodontal diseases
85	Diabetes mellitus promotes periodontal destruction in children	2007	Lalla E	Cheng B, Lal S, Kaplan S, Suavidade B, Greenberg E, Goland RS, Lamster IB	120 (8)	117 (7.8)	Columbia University	USA	America	Observational study	Journal of Clinical Periodontology (7.478)	Children; complications; diabetes; periodontitis
86	Association between type 1 and type 2 diabetes with periodontal disease and tooth loss	2009	Kaur G	Holtfreter B, Rathmann W, Schwahn C, Wallaschofski H, Schipf S, Nauck M, Kocher T	118 (9.07)	109 (8.38)	University of Greifswald	Germany	Europe	Observational study	Journal of Clinical Periodontology (7.478)	Attachment loss; epidemiology; periodontal disease; study of health in Pomerania; tooth loss; type 1 diabetes; type 2 diabetes
87	Effects of periodontal therapy on glycemic control and inflammatory markers	2008	O’Connell PA	Taba M, Nomizo A, Freitas MCF, Suaid FA, Uyemura SA, Trevisan GL, Novaes AB, Souza SLS, Palioto DB, Grisi MFM	118 (8.43)	114 (8.14)	University of São Paulo	Brazil	America	Randomized controlled trial	Journal of Periodontology (4.494)	Biomarkers; diabetes; inflammation; periodontitis
88	Hormonal influences: effects of diabetes mellitus and endogenous female sex steroid hormones on the periodontium	2003	Mealey BL	Moritz AJ	118 (6.21)	108 (5.68)	Wilford Hall Medical Center	USA	America	Literature review	Periodontology 2000 (7.589)	n.a.
89	Association between diabetes mellitus/hyperglycaemia and peri-implant diseases: Systematic review and meta-analysis	2017	Monje A	Catena A, Borgnakke WS	117 (23.4)	111 (22.2)	University of Michigan	USA	America	Systematic review/meta-analysis	Journal of Clinical Periodontology (7.478)	Dental implants; diabetes complications; epidemiology; gestational diabetes; glycosylated; haemoglobin A; humans; review; systematic
90	Effect of periodontal treatment on metabolic control, systemic inflammation and cytokines in patients with type 2 diabetes	2010	Correa FO	Gonçalves D, Figueredo CM, Bastos AS, Gustafsson A, Orrico SR	117 (9.75)	107 (8.92)	São Paulo State University	Brazil	America	Observational study	Journal of Clinical Periodontology (7.478)	Diabetes mellitus; fibrinogen; glycaemic control; periodontal disease; therapy; tumour necrosis factor-alpha
91	Smoking, diabetes mellitus, periodontitis, and supportive periodontal treatment as factors associated with dental implant survival: a long-term retrospective evaluation of patients followed for up to 10 years	2010	Anner R	Grossmann Y, Anner Y, Levin L	116 (9.67)	107 (8.92)	Sheba Medical Center	Israel	Asia	Observational study	Implant Dentistry (2.454)	Tobacco; periodontitis; diabetes mellitus; supportive therapy; implant failure
92	Periodontal findings in elderly patients with non-insulin dependent diabetes mellitus	1998	Collin HL	Uusitupa M, Niskanen L, Kontturi-Närhi V, Markkanen H, Koivisto AM, Meurman JH	116 (4.83)	110 (4.58)	University of Kuopio	Finland	Europe	Observational study	Journal of Periodontology (4.494)	Diabetes mellitus, non-insulin dependent; periodontal diseases microbiology; periodontitis/microbiology; metabolic control
93	Glycemic control and implant stabilization in type 2 diabetes mellitus	2009	Oates TW	Dowell S, Robinson M, McMahan CA	115 (8.85)	108 (8.31)	University of Texas Health Science Center at San Antonio	USA	America	Observational study	Journal of Dental Research (8.924)	Implants; hyperglycemia; diabetes mellitus; resonance frequency analysis; implant stability
94	Dental endosseous implant assessments in a type 2 diabetic population: a prospective study	2000	Olson JW	Shernoff AF, Tarlow JL, Colwell JA, Scheetz JP, Bingham SF	115 (5.23)	108 (4.91)	University of Louisville	USA	America	Observational study	The International Journal of Oral & Maxillofacial Implants (2.804)	Dental implants; diabetes; multicenter study; prospective studies
95	Does periodontal care improve glycemic control? The Department of Veterans Affairs Dental Diabetes Study	2007	Jones JÁ	Miller DR, Wehler CJ, Rich SE, Krall-Kaye EA, McCoy LC, Christiansen CL, Rothendler JA, Garcia RI	114 (7.6)	113 (7.53)	Boston University	USA	America	Randomized controlled trial	Journal of Clinical Periodontology (7.478)	Clinical trial; diabetes; glycemic control; HbA1c; periodontal disease
96	Missing Teeth Predict Incident Cardiovascular Events, Diabetes, and Death	2015	Liljestrand JM	Havulinna AS	112 (16)	110 (15.71)	University of Helsinki	Finland	Europe	Observational study	Journal of Dental Research (8.924)	Periodontitis; tooth extraction; cardiovascular diseases; partially edentulous jaw; edentulous mouth; diabetes mellitus
97	Salivary interleukin-6, matrix metalloproteinase-8, and osteoprotegerin in patients with periodontitis and diabetes	2010	Costa PP	Trevisan GL, Macedo GO,	112 (9.33)	104 (8.67)	University of São Paulo	Brazil	America	Observational study	Journal of Periodontology (4.494)	Diabetes mellitus; hemoglobin A; glycosylated; inflammation; interleukin-6; periodontitis; saliva
98	The effect of periodontal therapy in diabetics. Results after 5 years	1996	Westfelt E	Rylander H, Blohmé G, Jonasson P, Lindhe J	112 (4.31)	106 (4.08)	University of Gothenburg	Sweden	Europe	Observational study	Journal of Clinical Periodontology (7.478)	Diabetes mellitus; control of diabetes; periodontal therapy; periodontal maintenance therapy
99	Relation between control of diabetes and gingival bleeding	1985	Ervasti T	Knuuttila M, Pohjamo L, Haukipuro K	112 (3.03)	108 (2.92)	University of Oulu	Finland	Europe	Observational study	Journal of Periodontology (4.494)	n.a.
100	Periodontal disease experience in adult long-duration insulin-dependent diabetics	1993	Thorstensson H	Hugoson A	111 (3.83)	106 (3.66)	Jönköping University	Sweden	Europe	Observational study	Journal of Clinical Periodontology (7.478)	Diabetes-mellitus; insulin-dependent; periodontal disease

*mean based on the ratio of the numbers of citations and the period since the year of publication up to October 2022; n.a.: not applicable; n.i.: not informed; JCR® IF: Journal Citation Report Impact Factor.

The most-cited papers received a total of 18,694 citations (minimum: 111 citations; maximum: 566 citations) in WoS-AD, and 17,317 citations (minimum: 104 citations; maximum: 532 citations) in WoS-CC. The paper with the highest citation count^19^ was cited 566 times and had an average of 35.37 citations per year according to WoS-AD. This paper was also the most cited in WoS-CC, with 532 citations.

### Journal and year of publication

Overall, the papers in the top 100 list were published across 16 journals. The top four journals were the Journal of Periodontology (30%; JCR^®^ IF2021 – 4.494), the Journal of Clinical Periodontology (27%; JCR^®^ IF2021 – 7.478), the Journal of Dental Research (11%; JCR^®^ IF2021 – 8.924), and Periodontology 2000 (6%; JCR^®^ IF2021 – 7.589) (Table 1).

**Figure 1:**
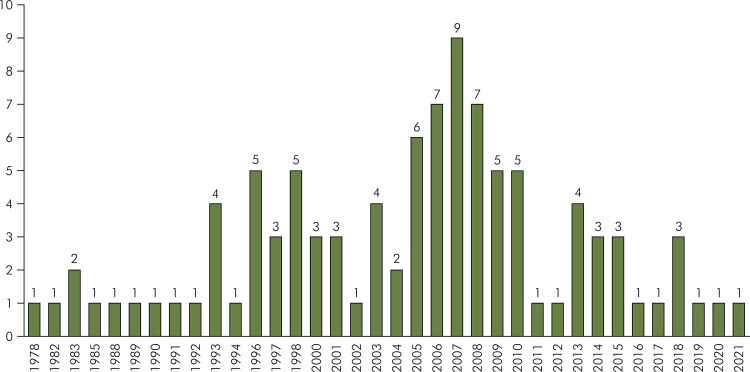
The number of publications of the 100 most-cited papers per year.

The selected papers were published between the years 1978 and 2021 (Table 1 and Figure 1). The half-decade of 2005 to 2010 exhibited the highest number of most-cited articles (n = 39), with a peak in 2007 (n = 9). The oldest and newest papers within the top 100 were observational studies. The oldest study in the top 100 list, published in 1978 by Basker et al.,^20^ has been cited 154 times (average of 3.4 citations per year). The most recent study, published in 2021 by Moorthy et al.,^21^ has been cited 124 times (average of 17.33 citations per year).

### Contributing authors

**Figure 2:**
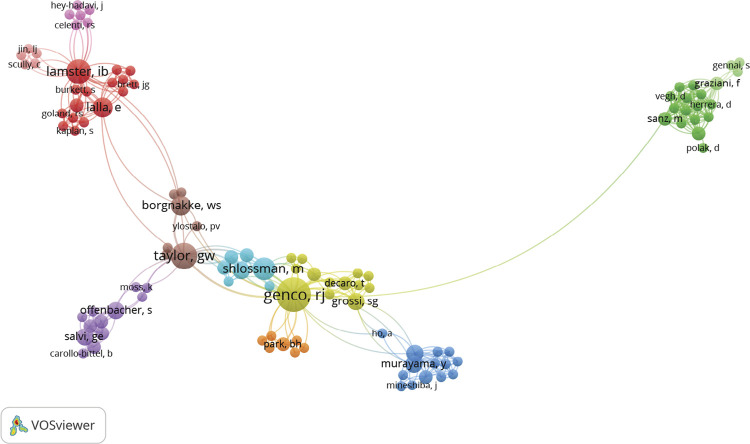
Co-authorship network map in top 100 most-cited papers on diabetes research in Dentistry.

Well-differentiated clusters are shown in the co-authorship network map (Figure 2), highlighting prominent research groups led by American researchers such as Genco, Taylor, Lamster, and Shlossman. Publications with three or more authors were more prevalent. Among the 374 authors identified, those with the most publications as first authors were Brian Mealey (n = 4; 1,138 citations), George Taylor (n = 3; 907 citations), Giovanni Salvi (n = 3; 497 citations), and Toshiyuki Saito (n = 3; 460 citations). However, Robert Genco was the author with the most significant contribution to the top 100 list, having authored 13 papers with a total of 3,653 citations. Following Genco, George Taylor ranked second with seven papers and 1,936 citations.

### Contributing institutions and countries

The articles originated from 54 institutions and were affiliated with 15 countries, identified through the corresponding author’s institution. The University at Buffalo (1,844 citations) and the University of Michigan (1,674 citations) emerged as the most prominent contributors, each with six publications. They were followed by the University of Texas (n = 5; 1,185 citations) and Columbia University (n = 5; 809 citations). Three papers did not provide information on the corresponding authors’ institutions.^22-24^

**Figure 3:**
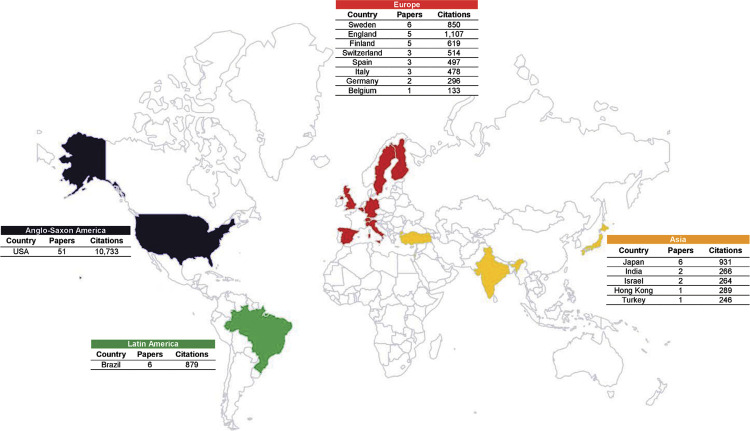
Global distribution of the top 100 most-cited papers on diabetes mellitus in Dentistry.

In terms of the countries of origin, the United States of America (USA) led with the highest number of publications and citations (n = 51; 10,733 citations), followed by Japan (n = 6; 931 citations), Sweden (n = 6; 850 citations), England (n = 5; 1,107 citations), Brazil (n = 5; 879 citations), and Finland (n = 5; 619 citations). No papers from Africa and Oceania were included in the list (Figure 3).

### Study design

Among the top 100 most-cited papers, 50 were observational studies (22,206 citations), 26 were literature reviews (5,209 citations), and 10 were systematic reviews (1,819 citations), with six including meta-analysis and four without (Table 2). Randomized and non-randomized clinical trials, laboratory studies, and case reports were less frequent in comparison.

**Table 2:** Characteristics of the 100 most-cited papers on diabetes research in Dentistry regarding study design.

Study design	Number of papers	Number of citations (WoS-AD)	Citation ratio*
Observational study	50	22206	444.1
Literature review	26	5209	200.3
Systematic review/meta-analysis	10	1819	181.9
*In vivo* (laboratory study)	6	1,195	199.2
Randomized controlled trial	5	914	182.8
Non-randomized clinical study	2	331	165.5
Case report	1	167	167
*In vitro* (laboratory study)	1	141	141

*Number of citations/number of papers; WoS-AD: Web of Science - All Databases.

### Keywords and research topic

**Figure 4:**
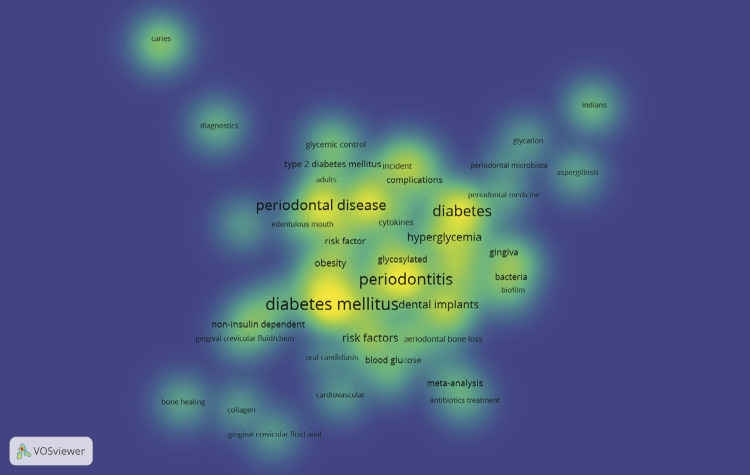
VOSviewer density map of co-occurrence of keywords.

Figure 4 shows a density map of connected keywords. A total of 233 keywords were identified, with 169 appearing only once. The term “diabetes mellitus” (n = 31) was the most frequently mentioned, followed by “periodontitis” (n = 26), “diabetes” (n = 24), “periodontal disease” (n = 19), “epidemiology” (n = 14), “hyperglycemia” (n = 10), and “inflammation” (n = 10).

Regarding the primary focus of the studies (Table 3), papers examining the relationship between DM and the periodontal disease were the most prevalent and achieved the highest citation count (n = 72; 14,077 citations). This was followed by research on the impact of DM on implant survival and/or osseointegration (n = 17; 2,399 citations). Only one included paper investigated the association between coronavirus disease and the occurrence of oral infection in patients with diabetes (124 citations).

**Table 3:** Main objective of the top 100 most-cited papers on diabetes mellitus in Dentistry.

Rank	References	Main objective
1	Mealey BL, Oates TW. American Academy of Periodontology. Diabetes mellitus and periodontal diseases. J Periodontol. 2006 Aug;77(8):1289-1303. https://doi.org/10.1902/jop.2006.050459	To review the literature concerning the relationship between DM and periodontal diseases.
2	Chapple IL, Genco R. Working group 2 of the joint EFP/AAP workshop. Diabetes and periodontal diseases: consensus report of the Joint EFP/AAP Workshop on Periodontitis and Systemic Diseases. J Periodontol. 2013 Apr;84(4):106-112. https://doi.org/10.1902/jop.2013.1340011.	To report the epidemiological evidence from cross-sectional, prospective and intervention studies for the impact of periodontal disease on diabetes incidence, control, and complications and to identify potential underpinning mechanisms.
3	King GL. The role of inflammatory cytokines in diabetes and its complications. J Periodontol. 2008 Aug;79(8):1527-34. https://doi.org/10.1902/jop.2008.080246.	To provide an understanding of the inflammatory basis for diabetes and its complications.
4	Haber J, Wattles J, Crowley M, Mandell R, Joshipura K, Kent RL. Evidence for cigarette smoking as a major risk factor for periodontitis. J Periodontol. 1993 Jan;64(1):16-23. https://doi.org/10.1902/jop.1993.64.1.16.	To evaluate the role of smoking as a risk factor for Periodontitis in diabetic and nondiabetic study groups.
5	Genco RJ, Grossi SG, Ho A, Nishimura F, Murayama Y. A Proposed Model Linking Inflammation to Obesity, Diabetes, and Periodontal Infections. J Periodontol. 2005 Nov;76 (11):2075-2084. https://doi.org/10.1902/jop.2005.76.11-S.2075.	To evaluate the relationship between obesity, periodontal disease, and DM insulin resistance as well as the plasma levels of TNFα and its soluble receptors to assess the relationship of inflammation to obesity, diabetes, and periodontal infections.
6	Emrich LJ, Shlossman M, Genco RJ. Periodontal disease in non-insulin-dependent diabetes mellitus. J Periodontol. 1991 Feb;62(2):123-31. https://doi.org/10.1902/jop.1991.62.2.123.	To determine the relationship between diabetes mellitus and oral health status in Pima Indians from the Gila River Indian Community in Arizona.
7	Taylor GW, Burt BA, Becker MP, Genco RJ, Shlossman M, Knowler WC, Pettitt DJ. Severe Periodontitis and Risk for Poor Glycemic Control in Patients with Non-Insulin-Dependent Diabetes Mellitus. J Periodontol. 1996 Oct;67 (10):1085-1093. https://doi.org/10.1902/jop.1996.67.10s.1085.	To test the hypothesis that severe periodontitis in persons with NIDDM increases the risk of poor glycemic control.
8	Golub LM, Lee HM, Lehrer G, Nemiroff A, McNamara TF, Kaplan R, Ramamurthy NS. Minocycline reduces gingival collagenolytic activity during diabetes. Preliminary observations and a proposed new mechanism of action. J Periodontal Res. 1983 Sep;18(5):516-26. https://doi.org/10.1111/j.1600-0765.1983.tb00388.x.	To describe initial studies using minocycline in diabetic rats and humans.
9	Taylor GW, Borgnakke WS. Periodontal disease: associations with diabetes, glycemic control and complications. Oral Dis. 2008 Apr;14(3):191-203. https://doi.org/10.1111/j.1601-0825.2008.01442.x.	To review the evidence for adverse effects of diabetes on periodontal health and periodontal disease on glycemic control and complications of diabetes.
10	Grossi SG, Skrepcinski FB, DeCaro T, Robertson DC, Ho AW, Dunford RG, Genco RJ. Treatment of periodontal disease in diabetics reduces glycated hemoglobin. J Periodontol. 1997 Aug;68(8):713-719. https://doi.org/10.1902/jop.1997.68.8.713.	To assess the effects of treatment of periodontal disease on the level of metabolic control of diabetes.
11	Tsai C, Hayes C, Taylor GW. Glycemic control of type 2 diabetes and severe periodontal disease in the US adult population. Community Dent Oral Epidemiol. 2002 Jun;30(3):182-192. https://doi.org/10.1034/j.1600-0528.2002.300304.x.	To investigate the association between glycemic control of T2DM and severe periodontal disease in the US adult population ages 45 years and older.
12	Borgnakke WS, Ylöstalo PV, Taylor GW, Genco RJ. Effect of periodontal disease on diabetes: systematic review of epidemiologic observational evidence. J Clin Periodontol. 2013 Apr;40 (14):135-52. https://doi.org/10.1111/jcpe.12080.	To systematically review non-experimental, epidemiologic evidence for effects of periodontal disease on diabetes control, complications, and incidence.
13	Jin LJ, Lamster IB, Greenspan JS, Pitts NB, Scully C, Warnakulasuriya S. Global burden of oral diseases: emerging concepts, management and interplay with systemic health. Oral Dis. 2016 Oct;22(7):609-619. https://doi.org/10.1111/odi.12428.	To present the global burden of major oral diseases with an exegetical commentary on their current profiles, the critical issues in oral healthcare and future perspectives.
14	Cianciola LJ, Park BH, Bruck E, Mosovich L, Genco RJ. Prevalence of periodontal disease in insulin-dependent diabetes mellitus (juvenile diabetes). J Am Dent Assoc. 1982 May;104(5):653-660. https://doi.org/10.14219/jada.archive.1982.0240.	To assess the correlation of the prevalence and severity of periodontal disease in the IDDM group with other factors, including supragingival dental plaque, age, and onset and duration of DM.
15	Mealey BL, Ocampo GL. Diabetes mellitus and periodontal disease. Periodontol 2000. 2007;44:127-153. https://doi.org/10.1111/j.1600-0757.2006.00193.x.	To review concepts and epidemiology of diabetes and its relationship with periodontal disease.
16	Kiran M, Arpak N, Unsal E, Erdoğan MF. The effect of improved periodontal health on metabolic control in type 2 diabetes mellitus. J Clin Periodontol. 2005 Mar;32(3):266-272. https://doi.org/10.1111/j.1600-051X.2005.00658.x.	To investigate the effect of improved periodontal health on metabolic control in T2DM patients.
17	Sanz M, Ceriello A, Buysschaert M, Chapple I, Demmer RT, Graziani F, Herrera D, Jepsen S, Lione L, Madianos P, Mathur M, Montanya E, Shapira L, Tonetti M, Vegh D. Scientific evidence on the links between periodontal diseases and diabetes: Consensus report and guidelines of the joint workshop on periodontal diseases and diabetes by the International Diabetes Federation and the European Federation of Periodontology. J Clin Periodontol. 2018 Feb;45(2):138-149. https://doi.org/10.1111/jcpe.12808.	To update the evidence for their epidemiological and mechanistic associations and re-examine the impact of effective periodontal therapy upon metabolic control (HbA1C).
18	Lamster IB, Lalla E, Borgnakke WS, Taylor GW. The relationship between oral health and diabetes mellitus. J Am Dent Assoc. 2008 Oct;139:19-24. https://doi.org/10.14219/jada.archive.2008.0363.	To review the literature to identify oral conditions that are affected by diabetes mellitus. To examine the literature concerning periodontitis as a modifier of glycemic control.
19	Chávarry NG, Vettore MV, Sansone C, Sheiham A. The relationship between diabetes mellitus and destructive periodontal disease: a meta-analysis. Oral Health Prev Dent. 2009;7(2):107-127.	To systematically review the studies on the association between diabetes mellitus and destructive periodontal disease.
20	Shlossman M, Knowler WC, Pettitt DJ, Genco RJ. Type 2 diabetes mellitus and periodontal disease. J Am Dent Assoc. 1990 Oct;121(4):532-536. https://doi.org/10.14219/jada.archive.1990.0211.	To evaluate the relationship between T2DM and periodontal disease.
21	Belibasakis GN, Bostanci N. The RANKL-OPG system in clinical periodontology. J Clin Periodontol. 2012 Mar;39(3):239-248. https://doi.org/10.1111/j.1600-051X.2011.01810.x.	To elaborate the current knowledge on RANKL and OPG in periodontal disease, and to evaluate their diagnostic and prognostic potential as biomarkers of the disease.
22	Javed F, Näsström K, Benchimol D, Altamash M, Klinge B, Engström PE. Comparison of periodontal and socioeconomic status between subjects with type 2 diabetes mellitus and non-diabetic controls. J Periodontol. 2007 Nov;78(11):2112-2119. https://doi.org/10.1902/jop.2007.070186.	To compare the periodontal conditions and socioeconomic status between subjects with T2DM and non-diabetic controls.
23	Javed F, Romanos GE. Impact of diabetes mellitus and glycemic control on the osseointegration of dental implants: a systematic literature review. J Periodontol. 2009 Nov;80(11):1719-1730. https://doi.org/10.1902/jop.2009.090283.	To assess the effects of diabetes and glycemic control on the osseointegration of dental implants.
24	Liu R, Bal HS, Desta T, Krothapalli N, Alyassi M, Luan Q, Graves DT. Diabetes enhances periodontal bone loss through enhanced resorption and diminished bone formation. J Dent Res. 2006 Jun;85(6):510-514. https://doi.org/10.1177/154405910608500606.	To investigate whether diabetes primarily affects periodontitis by enhancing bone loss or by limiting osseous repair.
25	Janket SJ, Wightman A, Baird AE, Van Dyke TE, Jones JA. Does periodontal treatment improve glycemic control in diabetic patients? A meta-analysis of intervention studies. J Dent Res. 2005 Dec;84(12):1154-1159. https://doi.org/10.1177/154405910508401212.	To review all published evidence systematically and to quantify the impact of periodontal treatment on HbA1c.
26	Grossi SG, Skrepcinski FB, DeCaro T, Zambon JJ, Cummins D, Genco RJ. Response to periodontal therapy in diabetics and smokers. J Periodontol. 1996 Oct;67(10):1094-1102. https://doi.org/10.1902/jop.1996.67.10s.1094.	To present the results of two independent clinical trials involving treatment of periodontal disease in diabetics and smokers.
27	Schmidt AM, Weidman E, Lalla E, Yan SD, Hori O, Cao R, Brett JG, Lamster IB. Advanced glycation endproducts (AGEs) induce oxidant stress in the gingiva: a potential mechanism underlying accelerated periodontal disease associated with diabetes. J Periodontal Res. 1996 Oct;31(7):508-515. https://doi.org/10.1111/j.1600-0765.1996.tb01417.x.	To establish the effects of infused AGEs on normal gingiva in vivo as well as to evaluate the AGEs levels and markers of oxidant stress in gingivafrom streptoztocin induced diabetic mice and from diabetic human subjects.
28	Salvi GE, Yalda B, Collins JG, Jones BH, Smith FW, Arnold RR, Offenbacher S. Inflammatory mediator response as a potential risk marker for periodontal diseases in insulin-dependent diabetes mellitus patients. J Periodontol. 1997 Feb;68(2):127-135. https://doi.org/10.1902/jop.1997.68.2.127.	To measure the gingival crevicular fluid and monocytic secretion of PGE2 and IL-1 beta in a group of IDDM patients and systemically healthy individuals.
29	Taylor GW, Burt BA, Becker MP, Genco RJ, Shlossman M, Knowler WC, Pettitt DJ. Non-insulin dependent diabetes mellitus and alveolar bone loss progression over 2 years. J Periodontol. 1998 Jan;69(1):76-83. https://doi.org/10.1902/jop.1998.69.1.76.	To evaluate if persons with NIDDM have greater risk of more severe alveolar bone loss progression than do those without NIDDM over a 2-year period.
30	Iwamoto Y, Nishimura F, Nakagawa M, Sugimoto H, Shikata K, Makino H, Fukuda T, Tsuji T, Iwamoto M, Murayama Y. The effect of antimicrobial periodontal treatment on circulating tumor necrosis factor-alpha and glycated hemoglobin level in patients with type 2 diabetes. J Periodontol. 2001 Jun;72(6):774-778. https://doi.org/10.1902/jop.2001.72.6.774.	To understand the effects of antimicrobial periodontal therapy on serum TNF-alpha concentration and subsequent metabolic control of diabetes.
31	Mealey BL. Periodontal disease and diabetes. A two-way street. J Am Dent Assoc. 2006 Oct;137: 26-31. https://doi.org/10.14219/jada.archive.2006.0404. Erratum in: J Am Dent Assoc. 2008 Mar;139(3):252.	To review the bidirectional relationships between diabetes and periodontal diseases.
32	Stewart JE, Wager KA, Friedlander AH, Zadeh HH. The effect of periodontal treatment on glycemic control in patients with type 2 diabetes mellitus. J Clin Periodontol. 2001 Apr;28(4):306-310. https://doi.org/10.1034/j.1600-051x.2001.028004306.x.	To explore the effect of periodontal therapy on glycemic control in persons with type 2 diabetes mellitus.
33	Nibali L, D’Aiuto F, Griffiths G, Patel K, Suvan J, Tonetti MS. Severe periodontitis is associated with systemic inflammation and a dysmetabolic status: a case-control study. J Clin Periodontol. 2007 Nov;34(11):931-937. https://doi.org/10.1111/j.1600-051X.2007.01133.x.	To investigate the association between severe periodontitis and increase in inflammatory and metabolic risk factors for cardiovascular disease.
34	Lösche W, Karapetow F, Pohl A, Pohl C, Kocher T. Plasma lipid and blood glucose levels in patients with destructive periodontal disease. J Clin Periodontol. 2000 Aug;27(8):537-541. https://doi.org/10.1034/j.1600-051x.2000.027008537.x.	To evaluate measure fasting plasma lipids as well as blood glucose in non-diabetic periodontal disease patients and control subjects.
35	Daubert DM, Weinstein BF, Bordin S, Leroux BG, Flemming TF. Prevalence and predictive factors for peri-implant disease and implant failure: a cross-sectional analysis. J Periodontol. 2015 Mar;86(3):337-347. https://doi.org/10.1902/jop.2014.140438.	To identify possible risk factors for implant loss and peri-implant diseases and to use those risk factors to form a predictive model for peri-implantitis and implant loss. It is also the aim to quantify the prevalence of peri-implant disease at ≈10 years after implant placement by using the best-available definitions of peri-implant diseases at the time of publication.
36	Moore PA, Guggenheimer J, Etzel KR, Weyant RJ, Orchard T. Type 1 diabetes mellitus, xerostomia, and salivary flow rates. Oral Surg Oral Med Oral Pathol Oral Radiol Endod. 2001 Sep;92(3):281-291. https://doi.org/10.1067/moe.2001.117815.	To describe the prevalence of dry-mouth symptoms (xerostomia), the prevalence of hyposalivation in this population, and the possible interrelationships between salivary dysfunction and diabetic complications
37	Bullon P, Newman HN, Battino M. Obesity, diabetes mellitus, atherosclerosis and chronic periodontitis: a shared pathology via oxidative stress and mitochondrial dysfunction? Periodontol 2000. 2014 Feb;64(1):139-153. https://doi.org/10.1111/j.1600-0757.2012.00455.x.	To review the literature to consider and discuss the mounting evidence that the basis for the inter-relationships between chronic periodontitis and atheromatous disease and diabetes lie at a fundamental intracellular level, namely oxidative stress, and mitochondrial dysfunction, as a meeting background among such chronic diseases and periodontitis.
38	Nishimura F, Iwamoto Y, Mineshiba J, Shimizu A, Soga Y, Murayama Y. Periodontal disease and diabetes mellitus: the role of tumor necrosis factor-alpha in a 2-way relationship. J Periodontol. 2003 Jan;74(1):97-102. https://doi.org/10.1902/jop.2003.74.1.97.	To review the literature concerning TNF-α produced by the adipose tissues of obese patients acts as a risk factor for periodontal inflammation, and TNF-α produced due to periodontal inflammation may be an additional important factor influencing insulin sensitivity in both obese and type 2 diabetic patients.
39	Rodrigues DC, Taba MJ, Novaes AB, Souza SL, Grisi MF. Effect of non-surgical periodontal therapy on glycemic control in patients with type 2 diabetes mellitus. J Periodontol. 2003 Sep;74(9):1361-1367. https://doi.org/10.1902/jop.2003.74.9.1361. Erratum in: J Periodontol. 2004 May;75(5):780.	To monitor the effect of non-surgical periodontal therapy on glycemic control in patients with T2DM.
40	Christgau M, Palitzsch KD, Schmalz G, Kreiner U, Frenzel S. Healing response to non-surgical periodontal therapy in patients with diabetes mellitus: clinical, microbiological, and immunologic results. J Clin Periodontol. 1998 Feb;25(2):112-124. https://doi.org/10.1111/j.1600-051x.1998.tb02417.x.	To monitor clinical, microbiological, medical, and immunological effects of non-surgical periodontal therapy in diabetics and healthy controls.
41	Engebretson S, Kocher T. Evidence that periodontal treatment improves diabetes outcomes: a systematic review and meta-analysis. J Periodontol. 2013 Apr;84(4):153-169. https://doi.org/10.1902/jop.2013.1340017	To evaluate the effect of periodontal treatment on diabetes outcomes.
42	Klokkevold PR, Han TJ. How do smoking, diabetes, and periodontitis affect outcomes of implant treatment? Int J Oral Maxillofac Implants. 2007;(22):173-202. Erratum in: Int J Oral Maxillofac Implants. 2008 Jan-Feb;23(1):56.	To evaluate the available literature to assess whether smoking, diabetes, and periodontitis have an adverse effect on the outcomes of implants placed in patients with these conditions.
43	Saito T, Shimazaki Y, Kiyohara Y, Kato I, Kubo M, Iida M, Yamashita Y. Relationship between obesity, glucose tolerance, and periodontal disease in Japanese women: the Hisayama study. J Periodontal Res. 2005 Aug;40(4):346-353. https://doi.org/10.1111/j.1600-0765.2005.00813.x.	To examine the relationship between obesity and periodontal disease by taking oral glucose tolerance test results into consideration.
44	Campus G, Salem A, Uzzau S, Baldoni E, Tonolo G. Diabetes and periodontal disease: a case-control study. J Periodontol. 2005 Mar;76(3):418-425. https://doi.org/10.1902/jop.2005.76.3.418.	To evaluate the possible association between NIDDM and clinical and microbiological periodontal disease among adult Sardinians.
45	Bullon P, Morillo JM, Ramirez-Tortosa MC, Quiles JL, Newman HN, Battino M. Metabolic syndrome and periodontitis: is oxidative stress a common link? J Dent Res. 2009 Jun;88(6):503-518. https://doi.org/10.1177/0022034509337479.	To analyze the published data to consider the hypothesis for a potential relationship between MetS and periodontitis, with oxidative stress acting as a putative link between both conditions.
46	Salvi GE, Carollo-Bittel B, Lang NP. Effects of diabetes mellitus on periodontal and peri-implant conditions: update on associations and risks. J Clin Periodontol. 2008 Sep;35(8):398-409. https://doi.org/10.1111/j.1600-051X.2008.01282.x.	To review the evidence for the association between diabetes and periodontal and peri-implant conditions and the impact of periodontal therapy in subjects with diabetes.
47	Graziani F, Gennai S, Solini A, Petrini M. A systematic review and meta-analysis of epidemiologic observational evidence on the effect of periodontitis on diabetes An update of the EFP-AAP review. J Clin Periodontol. 2018 Feb;45(2):167-187. https://doi.org/10.1111/jcpe.12837.	To update the available evidence on the impact of periodontitis on diabetes control, incidence and complications.
48	Casanova L, Hughes FJ, Preshaw PM. Diabetes and periodontal disease: a two-way relationship. Br Dent J. 2014 Oct;217(8):433-437. https://doi.org/10.1038/sj.bdj.2014.907.	To summarise our current understanding of the relationship between diabetes and periodontitis and to discuss the clinical implications of these findings for the dental professional.
49	Miller LS, Manwell MA, Newbold D, Reding ME, Rasheed A, Blodgett J, Kornman KS. The relationship between reduction in periodontal inflammation and diabetes control: a report of 9 cases. J Periodontol. 1992 Oct;63(10):843-848. https://doi.org/10.1902/jop.1992.63.10.843.	To evaluate the effect of controlling gingival inflammation on blood glucose levels as determined by glycosylation of hemoglobin and albumin.
50	Basker RM, Sturdee DW, Davenport JC. Patients with burning mouths. A clinical investigation of causative factors, including the climacteric and diabetes. Br Dent J. 1978 Jul 4;145(1):9-16. https://doi.org/10.1038/sj.bdj.4804107.	To investigate the profiles of burning mouth syndrome patients, its characteristics, and the available treatment methods and their effects.
51	Saito T, Shimazaki Y. Metabolic disorders related to obesity and periodontal disease. Periodontol 2000. 2007;43:254-66. https://doi.org/10.1111/j.1600-0757.2006.00186.x.	To review the relationship between obesity and periodontal disease and the causal relationship between obesity and diabetes
52	Tervonen T, Oliver RC. Long-term control of diabetes mellitus and periodontitis. J Clin Periodontol. 1993 Jul;20(6):431-435. https://doi.org/10.1111/j.1600-051x.1993.tb00384.x.	To evaluate the association between long-term control of diabetes mellitus (DM) and periodontitis.
53	Polak D, Shapira L. An update on the evidence for pathogenic mechanisms that may link periodontitis and diabetes. J Clin Periodontol. 2018 Feb;45(2):150-166. https://doi.org/10.1111/jcpe.12803.	To provide an update of the review by Taylor (Journal of Clinical Periodontology, 2013, 40, S113) regarding the scientific evidence of the biological association between periodontitis and diabetes.
54	Thorstensson H, Kuylenstierna J, Hugoson A. Medical status and complications in relation to periodontal disease experience in insulin-dependent diabetics. J Clin Periodontol. 1996 Mar;23(3):194-202. https://doi.org/10.1111/j.1600-051x.1996.tb02076.x.	To define a population of diabetics exhibiting an increased risk of developing severe periodontitis by comparing the medical status of 2 groups of diabetics, 1 with no/minor periodontal disease and 1 with severe periodontal disease.
55	Retzepi M, Donos N. The effect of diabetes mellitus on osseous healing. Clin Oral Implants Res. 2010 Jul;21(7):673-681. https://doi.org/10.1111/j.1600-0501.2010.01923.x.	To review the clinical evidence supporting a higher rate of complications during fracture healing in diabetic patients and the histological evidence indicating impaired potential for intramembranous and endochondral ossification in the presence of uncontrolled experimental diabetes.
56	Salvi GE, Collins JG, Yalda B, Arnold RR, Lang NP, Offenbacher S. Monocytic TNF alpha secretion patterns in IDDM patients with periodontal diseases. J Clin Periodontol. 1997 Jan;24(1):8-16. https://doi.org/10.1111/j.1600-051x.1997.tb01178.x.	To identify whether monocytic TNF alpha secretion patterns could serve as a potential phenotypic discriminator for periodontal disease susceptibility within IDDM patients.
57	Katz J, Bhattacharyya I, Farkhondeh-Kish F, Perez FM, Caudle RM, Heft MW. Expression of the receptor of advanced glycation end products in gingival tissues of type 2 diabetes patients with chronic periodontal disease: a study utilizing immunohistochemistry and RT-PCR. J Clin Periodontol. 2005 Jan;32(1):40-44. https://doi.org/10.1111/j.1600-051X.2004.00623.x.	To demonstrate the presence of RAGE in human periodontium in patients with chronic periodontitis with and without T2DM.
58	Saito T, Shimazaki Y, Kiyohara Y, Kato I, Kubo M, Iida M, Koga T. The severity of periodontal disease is associated with the development of glucose intolerance in non-diabetics: the Hisayama study. J Dent Res. 2004 Jun;83(6):485-490. https://doi.org/10.1177/154405910408300610.	To evaluate the relationship between periodontitis and glucose tolerance status, including changes in status.
59	Hugoson A, Thorstensson H, Falk H, Kuylenstierna J. Periodontal conditions in insulin-dependent diabetics. J Clin Periodontol. 1989 Apr;16(4):215-223. https://doi.org/10.1111/j.1600-051x.1989.tb01644.x.	To compare the prevalence and severity of periodontal disease in age- and sex-matched adult long- and short-duration IDDM and NIDDM.
60	Kumar MS, Vamsi G, Sripriya R, Sehgal PK. Expression of matrix metalloproteinases (MMP-8 and -9) in chronic periodontitis patients with and without diabetes mellitus. J Periodontol. 2006 Nov;77(11):1803-1808. https://doi.org/10.1902/jop.2006.050293.	To assess the expression of MMP-8 and -9 in gingival tissues of diabetic chronic periodontitis, non-diabetic chronic periodontitis, and healthy patients.
61	Oliver RC, Tervonen T. Diabetes-A Risk Factor for Periodontitis in Adults? J Periodontol. 1994 May;65 (5):530-538. https://doi.org/10.1902/jop.1994.65.5s.530.	To review variations in type, metabolic control, and duration of diabetes and highlights the results of studies that have considered these variations.
62	Zambon JJ, Reynolds H, Fisher JG, Shlossman M, Dunford R, Genco RJ. Microbiological and immunological studies of adult periodontitis in patients with noninsulin-dependent diabetes mellitus. J Periodontol. 1988 Jan;59(1):23-31. https://doi.org/10.1902/jop.1988.59.1.23.	To report an examination of the subgingival microflora and serum antibody response in noninsulin-dependent diabetes mellitus.
63	Kinane D, Bouchard P; Group E of European Workshop on Periodontology. Periodontal diseases and health: Consensus Report of the Sixth European Workshop on Periodontology. J Clin Periodontol. 2008 Sep;35(8):333-337. https://doi.org/10.1111/j.1600-051X.2008.01278.x.	To update the knowledge base on periodontal diseases and health.
64	Shimazaki Y, Saito T, Yonemoto K, Kiyohara Y, Iida M, Yamashita Y. Relationship of metabolic syndrome to periodontal disease in Japanese women: the Hisayama Study. J Dent Res. 2007 Mar;86(3):271-275. https://doi.org/10.1177/154405910708600314.	To examine the relationship between periodontitis and 5 components of metabolic syndrome--abdominal obesity, triglyceride level, high-density lipoprotein cholesterol level, blood pressure, and fasting blood sugar level--in 584 Japanese women.
65	Southerland JH, Taylor GW, Moss K, Beck JD, Offenbacher S. Commonality in chronic inflammatory diseases: periodontitis, diabetes, and coronary artery disease. Periodontol 2000. 2006;40:130-143. https://doi.org/10.1111/j.1600-0757.2005.00138.x.	To review the relationship between chronic inflammatory diseases: periodontitis, diabetes, and coronary artery disease.
66	Mombelli A, Cionca N. Systemic diseases affecting osseointegration therapy. Clin Oral Implants Res. 2006 Oct;17(2):97-103. https://doi.org/10.1111/j.1600-0501.2006.01354.x. Erratum in: Clin Oral Implants Res. 2006 Dec;17(6):746.	To evaluate the impact of systemic diseases and their treatment on the success of osseointegration therapy.
67	Nevins ML, Karimbux NY, Weber HP, Giannobile WV, Fiorellini JP. Wound healing around endosseous implants in experimental diabetes. Int J Oral Maxillofac Implants. 1998 Sep-Oct;13(5):620-629.	To identify the effects of streptozotocin-induced diabetes on osseointegration.
68	Alsaadi G, Quirynen M, Michiles K, Teughels W, Komárek A, van Steenberghe D. Impact of local and systemic factors on the incidence of failures up to abutment connection with modified surface oral implants. J Clin Periodontol. 2008 Jan;35(1):51-57. https://doi.org/10.1111/j.1600-051X.2007.01165.x.	To assess the influence of systemic and local bone and intra-oral factors on the occurrence of early TiUnite implant failures.
69	Navarro-Sanchez AB, Faria-Almeida R, Bascones-Martinez A. Effect of non-surgical periodontal therapy on clinical and immunological response and glycaemic control in type 2 diabetic patients with moderate periodontitis. J Clin Periodontol. 2007 Oct;34(10):835-843. https://doi.org/10.1111/j.1600-051X.2007.01127.x.	To compare the local efficacy of nonsurgical periodontal therapy between T2DM and non-diabetic patients and the effect of periodontal therapy on glycaemic control.
70	Casarin RC, Barbagallo A, Meulman T, Santos VR, Sallum EA, Nociti FH, Duarte PM, Casati MZ, Gonçalves RB. Subgingival biodiversity in subjects with uncontrolled type-2 diabetes and chronic periodontitis. J Periodontal Res. 2013 Feb;48(1):30-36. https://doi.org/10.1111/j.1600-0765.2012.01498.x.	To compare the subgingival biodiversity in deep periodontal pockets of subjects with chronic periodontitis and either uncontrolled T2DM or no diabetes using 16S rRNA gene cloning and sequencing.
71	Wu YY, Xiao E, Graves DT. Diabetes mellitus related bone metabolism and periodontal disease. Int J Oral Sci. 2015 Jun 26;7(2):63-72. https://doi.org/10.1038/ijos.2015.2.	To discuss the potential mechanism of diabetes-enhanced bone loss in relation to osteoblasts and osteoclasts.
72	Morita T, Yamazaki Y, Mita A, Takada K, Seto M, Nishinoue N, Sasaki Y, Motohashi M, Maeno M. A cohort study on the association between periodontal disease and the development of metabolic syndrome. J Periodontol. 2010 Apr;81(4):512-519. https://doi.org/10.1902/jop.2010.090594.	To investigate the association between periodontal disease and changes in metabolic-syndrome components to accumulate evidence of the causal relationship between the two conditions.
73	Chrcanovic BR, Albrektsson T, Wennerberg A. Diabetes and oral implant failure: a systematic review. J Dent Res. 2014 Sep;93(9):859-867. https://doi.org/10.1177/0022034514538820.	To investigate whether there are any effects of diabetes mellitus on implant failure rates, postoperative infections, and marginal bone loss.
74	Seppälä B, Seppälä M, Ainamo J. A longitudinal study on insulin-dependent diabetes mellitus and periodontal disease. J Clin Periodontol. 1993 Mar;20(3):161-165. https://doi.org/10.1111/j.1600-051x.1993.tb00338.x.	To assess the progression of periodontal disease after 1 year from the baseline examination in 38 dentate subjects and after 2 years in 22 dentate subjects with a mean duration of 18 years of insulin-dependent diabetes mellitus.
75	Graves DT, Corrêa JD, Silva TA. The Oral Microbiota Is Modified by Systemic Diseases. J Dent Res. 2019 Feb;98(2):148-156. https://doi.org/10.1177/0022034518805739.	To review the relationship systemic diseases such as diabetes, rheumatoid arthritis, and systemic lupus erythematosus in the increase susceptibility to destructive periodontal diseases.
76	Graves DT, Liu R, Alikhani M, Al-Mashat H, Trackman PC. Diabetes-enhanced inflammation and apoptosis--impact on periodontal pathology. J Dent Res. 2006 Jan;85(1):15-21. https://doi.org/10.1177/154405910608500103.	To review how diabetes-enhanced inflammation and apoptosis may affect the oral environment.
77	Engebretson SP, Hey-Hadavi J, Ehrhardt FJ, Hsu D, Celenti RS, Grbic JT, Lamster IB. Gingival crevicular fluid levels of interleukin-1beta and glycemic control in patients with chronic periodontitis and type 2 diabetes. J Periodontol. 2004 Sep;75(9):1203-1208. https://doi.org/10.1902/jop.2004.75.9.1203.	To determine whether glycemic control was related to gingival crevicular fluid levels of IL-1beta.
78	Guggenheimer J, Moore PA, Rossie K, Myers D, Mongelluzzo MB, Block HM, Weyant R, Orchard T. Insulin-dependent diabetes mellitus and oral soft tissue pathologies: II. Prevalence and characteristics of Candida and Candidal lesions. Oral Surg Oral Med Oral Pathol Oral Radiol Endod. 2000 May;89(5):570-576. https://doi.org/10.1067/moe.2000.104477.	To assess the prevalence of Candida albicans and oral infection with Candida in patients with insulin-dependent diabetes mellitus.
79	Moorthy A, Gaikwad R, Krishna S, Hegde R, Tripathi KK, Kale PG, Rao PS, Haldipur D, Bonanthaya K. SARS-CoV-2, Uncontrolled Diabetes and Corticosteroids-An Unholy Trinity in Invasive Fungal Infections of the Maxillofacial Region? A Retrospective, Multi-centric Analysis. J Maxillofac Oral Surg. 2021 Sep;20(3):418-425. https://doi.org/10.1007/s12663-021-01532-1.	To investigate the common contributing factors leading to such infections and of highlighting the significance of this surge seen in patients infected with SARS-CoV-2.
80	Mashimo PA, Yamamoto Y, Slots J, Park BH, Genco RJ. The periodontal microflora of juvenile diabetics. Culture, immunofluorescence, and serum antibody studies. J Periodontol. 1983 Jul;54(7):420-430. https://doi.org/10.1902/jop.1983.54.7.420.	To evaluate the composition of the subgingival flora in IDDM patients suffering from Periodontitis using cultural, phase contrast and immunofluorescent microscopic procedures.
81	Lalla E, Kunzel C, Burkett S, Cheng B, Lamster IB. Identification of unrecognized diabetes and pre-diabetes in a dental setting. J Dent Res. 2011 Jul;90(7):855-860. https://doi.org/10.1177/0022034511407069. Epub 2011 Apr 29. Erratum in: J Dent Res. 2012 Jul;91(7):715.	To assess the performance of a targeted approach to identify unrecognized diabetes and pre-diabetes in a population presenting at a dental clinic.
82	Fouad AF, Burleson J. The effect of diabetes mellitus on endodontic treatment outcome: data from an electronic patient record. J Am Dent Assoc. 2003 Jan;134(1):43-51; quiz 117-8. https://doi.org/10.14219/jada.archive.2003.0016.	To investigate endodontic diagnostic and treatment outcome data in patients with and without diabetes.
83	Scannapieco FA. Position paper of The American Academy of Periodontology: periodontal disease as a potential risk factor for systemic diseases. J Periodontol. 1998 Jul;69(7):841-850.	To provide information regarding the role of periodontal disease in systemic diseases, including bacteremia, infective endocarditis, cardiovascular disease and atherosclerosis, prosthetic device infection, diabetes mellitus, respiratory diseases, and adverse pregnancy outcomes.
84	Genco RJ, Sanz M. Clinical and public health implications of periodontal and systemic diseases: An overview. Periodontol 2000. 2020 Jun;83(1):7-13. https://doi.org/10.1111/prd.12344.	To describe the emerging evidence and updates the current state of knowledge regarding the associations between periodontal diseases, mainly periodontitis, and several systemic diseases.
85	Lalla E, Cheng B, Lal S, Kaplan S, Softness B, Greenberg E, Goland RS, Lamster IB. Diabetes mellitus promotes periodontal destruction in children. J Clin Periodontol. 2007 Apr;34(4):294-298. https://doi.org/10.1111/j.1600-051X.2007.01054.x.	To assess the periodontal status of a large cohort of children and adolescents with diabetes.
86	Kaur G, Holtfreter B, Rathmann W, Schwahn C, Wallaschofski H, Schipf S, Nauck M, Kocher T. Association between type 1 and type 2 diabetes with periodontal disease and tooth loss. J Clin Periodontol. 2009 Sep;36(9):765-774. https://doi.org/10.1111/j.1600-051X.2009.01445.x.	To determine whether both T1DM and T2DM are associated with increased prevalence and extent of periodontal disease and tooth loss compared with non-diabetic subjects within a homogeneous adult study population.
87	O’Connell PA, Taba M, Nomizo A, Foss Freitas MC, Suaid FA, Uyemura SA, Trevisan GL, Novaes AB, Souza SL, Palioto DB, Grisi MF. Effects of periodontal therapy on glycemic control and inflammatory markers. J Periodontol. 2008 May;79(5):774-783. https://doi.org/10.1902/jop.2008.070250.	To evaluate the effects of periodontal therapy on the serum levels of HbA1c and on inflammatory biomarkers.
88	Mealey BL, Moritz AJ. Hormonal influences: effects of diabetes mellitus and endogenous female sex steroid hormones on the periodontium. Periodontol 2000. 2003;32:59-81. https://doi.org/10.1046/j.0906-6713.2002.03206.x.	To discuss the primary hormonal factors associated with diabetes mellitus and female sex steroid hormones.
89	Monje A, Catena A, Borgnakke WS. Association between diabetes mellitus/hyperglycaemia and peri-implant diseases: Systematic review and meta-analysis. J Clin Periodontol. 2017 Jun;44(6):636-648. https://doi.org/10.1111/jcpe.12724.	To investigate whether hyperglycaemia/DM is associated with peri-implant diseases (peri-implant mucositis and peri-implantitis).
90	Correa FO, Gonçalves D, Figueredo CM, Bastos AS, Gustafsson A, Orrico SR. Effect of periodontal treatment on metabolic control, systemic inflammation and cytokines in patients with type 2 diabetes. J Clin Periodontol. 2010 Jan;37(1):53-58. https://doi.org/10.1111/j.1600-051X.2009.01498.x.	To investigate the effect of periodontal therapy on the circulating concentration of hs-CRP, FIB, IL-4, IL-6, IL-8, IL-10 and TNF-alpha and on the metabolic control in T2DM patients.
91	Anner R, Grossmann Y, Anner Y, Levin L. Smoking, diabetes mellitus, periodontitis, and supportive periodontal treatment as factors associated with dental implant survival: a long-term retrospective evaluation of patients followed for up to 10 years. Implant Dent. 2010 Feb;19(1):57-64. https://doi.org/10.1097/ID.0b013e3181bb8f6c.	To evaluate the factors associated with long-term implant survival in a large cohort of patients in regular follow-up until data collection.
92	Collin HL, Uusitupa M, Niskanen L, Kontturi-Närhi V, Markkanen H, Koivisto AM, Meurman JH. Periodontal findings in elderly patients with non-insulin dependent diabetes mellitus. J Periodontol. 1998 Sep;69(9):962-966. https://doi.org/10.1902/jop.1998.69.9.962.	To investigate periodontal status, including assessment of 3 putative periodontal pathogens, in a group of elderly Finnish patients with non-insulin-dependent diabetes mellitus.
93	Oates TW, Dowell S, Robinson M, McMahan CA. Glycemic control and implant stabilization in type 2 diabetes mellitus. J Dent Res. 2009 Apr;88(4):367-371. https://doi.org/10.1177/0022034509334203.	To evaluate the effect of glycemic level on implant integration in persons with diabetes
94	Olson JW, Shernoff AF, Tarlow JL, Colwell JA, Scheetz JP, Bingham SF. Dental endosseous implant assessments in a type 2 diabetic population: a prospective study. Int J Oral Maxillofac Implants. 2000 Nov-Dec;15(6):811-818.	To assess the success of 2-stage endosseous root-form implants (3 different implant systems) placed in the mandibular symphysis of 89 male type 2 diabetic subjects.
95	Jones JA, Miller DR, Wehler CJ, Rich SE, Krall-Kaye EA, McCoy LC, Christiansen CL, Rothendler JA, Garcia RI. Does periodontal care improve glycemic control? The Department of Veterans Affairs Dental Diabetes Study. J Clin Periodontol. 2007 Jan;34(1):46-52. https://doi.org/10.1111/j.1600-051X.2006.01002.x.	To report the efficacy of periodontal care in the improvement of glycemic control in 165 veterans with poorly controlled diabetes over 4 mo.
96	Liljestrand JM, Havulinna AS, Paju S, Männistö S, Salomaa V, Pussinen PJ. Missing Teeth Predict Incident Cardiovascular Events, Diabetes, and Death. J Dent Res. 2015 Aug;94(8):1055-1062. https://doi.org/10.1177/0022034515586352.	To study the capability of the number of missing teeth in predicting incident cardiovascular diseases, diabetes, and all-cause death.
97	Costa PP, Trevisan GL, Macedo GO, Palioto DB, Souza SL, Grisi MF, Novaes AB Jr, Taba M Jr. Salivary interleukin-6, matrix metalloproteinase-8, and osteoprotegerin in patients with periodontitis and diabetes. J Periodontol. 2010 Mar;81(3):384-391. https://doi.org/10.1902/jop.2009.090510.	To evaluate the salivary concentrations of IL-6, MMP-8, and OPG in patients with periodontitis withT2DM.
98	Westfelt E, Rylander H, Blohmé G, Jonasson P, Lindhe J. The effect of periodontal therapy in diabetics. Results after 5 years. J Clin Periodontol. 1996 Feb;23(2):92-100. https://doi.org/10.1111/j.1600-051x.1996.tb00540.x.	To study the frequency of recurrence of periodontitis in diabetic subjects, who, prior to the initiation of a 5-year period of monitoring, were treated for moderate to advanced periodontal disease.
99	Ervasti T, Knuuttila M, Pohjamo L, Haukipuro K. Relation between control of diabetes and gingival bleeding. J Periodontol. 1985 Mar;56(3):154-157. https://doi.org/10.1902/jop.1985.56.3.154.	To examine the periodontal health status of adult diabetics and healthy controls.
100	Thorstensson H, Hugoson A. Periodontal disease experience in adult long-duration insulin-dependent diabetics. J Clin Periodontol. 1993 May;20(5):352-358. https://doi.org/10.1111/j.1600-051x.1993.tb00372.x.	To analyse periodontal disease experience in 40- to 70-year-old, sex-matched IDDM and NIDDM.

AGEs: advanced glycation endoproducts; DM: diabetes mellitus; FIB: fibrinogen; hs-CRP: HbA1c: Hemoglobin A1C; IL: interleukin; IDDM: insulin-dependent diabetes mellitus; high-sensitivity capsule-reactive protein; mo: months; MMP: matrix metalloproteinase; NIDDM: non-insulin dependent diabetes mellitus; OPG: osteoprotegerin; PGE2: prostaglandin E2; RANKL: receptor activator of nuclear factor kappa beta; TNF: tumour necrosis factor; T1DM: type 1 diabetes mellitus; T2DM: type 2 diabetes mellitus; US: United States.

## Discussion

The emerging scientific evidence indicates a bidirectional association between DM and oral conditions, with each impacting the other mutually.^25^ As a prevalent metabolic disease among patients, the relationship between DM and oral health has emerged as a promising area of study within both medical^26^ and dental research.^27^ In this regard, this is the first bibliometric study to access the features of the 100 most-cited papers on DM research published in Dentistry. The most-cited papers in this bibliometric analysis received 18,694 in WoS-AD, with individual citations ranging from 111 to 566.

The existing literature suggests that classic papers typically have at least 400 citations, serving as key references in the advancement of research and clinical practice.^28^ Depending on the specific characteristics of the research area, articles with at least 100 citations can also be considered classics.^29,30^ In this bibliometric analysis, the paper ranked at position 100 had 111 citations in WoS-AD. Therefore, it is reasonable to infer that all selected papers have made a significant impact on the field of diabetes research in dental journals. This feature not only demonstrates the importance of the theme over the years but also implies that other “classic articles” might have been omitted.

When the papers were ranked in descending order of citation number, the literature review “Diabetes mellitus and periodontal diseases” written by Mealey & Oates^31^ was ranked first. Besides citation count, other important aspects should be considered when evaluating the scientific impact of a paper, such as the methodological quality and study design.^30^ The most-cited paper received fewer citations per year compared to more recent papers that were ranked lower. For instance, an umbrella review published in 2020,^32^ ranked 84^th^, received more citations per year than the aforementioned article.^31^ Similarly, systematic reviews were the third most prevalent study design on the list, with six studies being systematic reviews complemented by meta-analysis, representing the top of scientific evidence. Thus, this higher citation density may be related to the topic and study design. Therefore, evaluating the average number of citations received per year, as also considered in other bibliometric analyses,^30,33^ over citation count alone, may provide a more significant parameter for assessing the relevance of a study in a specific research area.

The 100 most-cited papers were published within a 45-year timeframe, which may not be considered a long period given the significant rise in the prevalence of DM in the population over the years^1^ and the extensive efforts to control DM over decades. Although the oldest paper was an observational study not entirely focused on DM,^20^ the authors conducted a causality analysis on burning mouth syndrome in diabetic patients, establishing an important relationship between the two conditions. However, the most influential papers were predominantly published in the 2000s, demonstrating the high interest in this research topic over the last 20 years. Additionally, more than half of all included studies (n = 68) were published in only three peer-reviewed journals: Journal of Periodontology, Journal of Clinical Periodontology, and Journal of Dental Research. These data confirm that leading journals in a research field tend to attract papers that are likely to have a high citation number, thereby maintaining the high impact factor of those journals.^34^

Based on the data collected from the selected papers, Periodontology emerges as the area that has most extensively investigated the relationship between DM and Dentistry. Conversely, areas such as Oral Pathology, Oral Implantology, and Endodontics were less frequently identified in the list. The most cited papers have predominantly explored the relationship between DM and periodontal disease in humans, showcasing a significant interest in understanding the influence and correlation of DM and other prevalent diseases on the progression of periodontal infections. Most of the research has focused on diagnosing and treating periodontal conditions in diabetic patients, yielding positive outcomes for the improvement of the oral and systemic health of affected individuals. Furthermore, papers evaluating the impact of DM on osseointegration and stability of dental implants were also frequently observed. According to these features, “diabetes mellitus” and “periodontitis” were the most used keywords among the most-cited papers. However, although the main topics of interest among the most-cited papers were primarily concerned with the evaluation of different aspects of DM on periodontal research, many keywords were used in a single paper (72.5%). This point indicates a lack of standardization in the use of these important components of bibliographic search. Unfortunately, several papers did not include keywords,^20,22-24,35-46^ underscoring the importance of authors using strategic terms in their titles and abstracts that are closely related to the area and topic of interest, thus facilitating the retrieval of more relevant results.

Overall, the greatest contribution was made by the American author Robert J. Genco, a pioneer in periodontal research, who also emerged as the most-cited author. George W. Taylor also played a crucial role in DM research, ranking second in terms of citations. Consequently, the State University of New York at Buffalo and the University of Michigan, the affiliations of these influential authors, emerged as the most prolific institutions in diabetes research in dentistry. In concordance with other bibliometric studies in diabetes,^14,15^ the USA and its academic institutions continue to have the largest contributions in this research field. The USA is renowned for its well–established and extensive scientific community, bolstered by widespread public support and significant investment from the US government in scientific research. Furthermore, similar to previous bibliometric studies in Dentistry,^17,18,47^ no papers from African countries were included in the list. This absence could be attributed to various factors, including language barriers, challenges in professional networking, and limited access to information.^47^

Considering the impact of different variables, such as demographic and economic factors, on the relationship between diabetes and oral health, few randomized clinical trials were identified among the most-cited papers in the current bibliometric analysis. Furthermore, this study has some limitations that need to be considered. Firstly, there is a possibility that some important articles without specific keywords or terms matching the current search strategy were not retrieved in the electronic search. Additionally, similar to other bibliometric studies published in Dentistry^48-53^ and other biomedical fields,^13,14,16^ only one database was used as the source of abstracts, citations, and other bibliometric data. The present analysis only included papers from the WoS citation indexing database, which is considered a limitation of this article. This database was selected because it covers over 34,000 high-quality and peer-reviewed journals in more than 250 areas,^54^ and has also measured citation numbers since 1950.^55^ However, other databases, such as Scopus and Google Scholar, should also be considered in future analysis. Finally, self-citations were not considered during the quantification of citations. Although self-citations may not significantly affect the order of the top 100 list,^33,56^ they should be interpreted with caution. Self-citations could also be a common practice in lines of investigation involving a limited number of researchers and may serve to save space by referencing methodologies that have been previously described elsewhere.^18,33^

Based on the data derived from the assessed features of this bibliometric analysis, further studies are encouraged on this topic, particularly in lower-income areas, using large-scale, randomized designs in community settings. Additionally, a more comprehensive examination of demographic and economic variables is warranted to determine their effect on the relationship between DM and oral condition in humans, especially across other areas of Dentistry.

## Conclusion

This bibliometric study provided useful data regarding the main features, direction, and most prolific research groups involved in DM research within Dentistry. The area of Periodontology stood out in the most cited studies that evaluated the relationship between Dentistry and DM, primarily originating from institutions such as the State University of New York at Buffalo and the University of Michigan in the USA. Observational studies exploring the relationship between DM and periodontal disease garnered the highest citation counts thus far.
